# Evidence for Functional Groupings of Vibrissae across the Rodent Mystacial Pad

**DOI:** 10.1371/journal.pcbi.1004109

**Published:** 2016-01-08

**Authors:** Jennifer A. Hobbs, R. Blythe Towal, Mitra J. Z. Hartmann

**Affiliations:** 1 Department of Physics and Astronomy, Northwestern University, Evanston, Illinois, United States of America; 2 Department of Biomedical Engineering, Northwestern University, Evanston, Illinois, United States of America; 3 Department of Mechanical Engineering, Northwestern University, Evanston, Illinois, United States of America; Queen's University, CANADA

## Abstract

During natural exploration, rats exhibit two particularly conspicuous vibrissal-mediated behaviors: they follow along walls, and “dab” their snouts on the ground at frequencies related to the whisking cycle. In general, the walls and ground may be located at any distance from, and at any orientation relative to, the rat’s head, which raises the question of how the rat might determine the position and orientation of these surfaces. Previous studies have compellingly demonstrated that rats can accurately determine the horizontal angle at which a vibrissa first touches an object, and we therefore asked whether this parameter could provide the rat with information about the pitch, distance, and yaw of a surface relative to its head. We used a three-dimensional model of the whisker array to construct mappings between the horizontal angle of contact of each vibrissa and every possible (pitch, distance, and yaw) configuration of the head relative to a flat surface. The mappings revealed striking differences in the patterns of contact for vibrissae in different regions of the array. The exterior (A, D, E) rows provide information about the relative pitch of the surface regardless of distance. The interior (B, C) rows provide distance cues regardless of head pitch. Yaw is linearly correlated with the difference between the number of right and left whiskers touching the surface. Compared to the long reaches that whiskers can make to the side and below the rat, the reachable distance in front of the rat’s nose is relatively small. We confirmed key predictions of these functional groupings in a behavioral experiment that monitored the contact patterns that the vibrissae made with a flat vertical surface. These results suggest that vibrissae in different regions of the array are not interchangeable sensors, but rather functionally grouped to acquire particular types of information about the environment.

## Author Summary

Animals do not passively sense the world. They actively move their sensors to acquire the information best suited to their current behavioral needs. Thus, to study neural processing in any sensory system, it is critical to determine the spatiotemporal structure of the inputs to that system. The rat whisker system is a well-studied model of active sensing because rats rhythmically brush and tap their vibrissae against objects during tactual exploration. To date, however, the patterns of sensory input during vibrisso-tactile exploration have not been quantified. This study quantifies some of the statistics of vibrissal-surface contact, focusing specifically on the angle of contact with a flat surface. Given that during exploration the rat could encounter a surface at any position and orientation relative to its head, we simulated the spatial patterns of vibrissal contact for all possible head-surface configurations. Results reveal functional groupings of whiskers across the array, with different whiskers being better suited for different aspects of tactile sensory tasks than others. Key predictions of the simulations were validated in a behavioral experiment that monitored vibrissal contact patterns with a flat wall. These results provide some of the first quantitative insights into the “natural tactile scene” for the vibrisso-trigeminal system.

## Introduction

Rodents use rhythmic movements of their large mystacial vibrissae (whiskers) to tactually explore objects in their environment [1,2]. During tactile exploration, rats exhibit two particularly conspicuous vibrissal-related behaviors: they tend to follow along walls and to “dab” their snouts on the ground and surfaces at frequencies related to the whisking and sniffing cycle [2–5].

Over the course of a whisk, the surface area formed by the vibrissal tips ranges approximately between 20 and 70 cm^2^, equivalent to a circle with a diameter between 5 and 10 cm [6,7]. When the rat encounters a surface (a wall or the ground) at this spatial scale, the surface can be located at any distance from, and at any orientation relative to, the rat’s head. This raises the obvious question of how the rat might use its whiskers to determine the position and orientation of the ground and surrounding walls without visual cues.

Answering this question through behavioral experiments would be a daunting task, requiring careful monitoring of both the head and vibrissae relative to a variety of ground and wall orientations. Instead, in the present study, we used a three-dimensional (3D) model of the rat head and vibrissal array to simulate vibrissal-object contact patterns with planar surfaces. These simulations allowed us to systematically explore the contact patterns of all 62 vibrissae over all possible head positions and orientations relative to a flat surface. We focused particularly on identifying the horizontal angle at which each vibrissa first touched the surface, because previous studies have compellingly demonstrated that rats can determine this angle with particularly high resolution [8,9].

The simulations revealed striking differences in the patterns of contact for vibrissae in different regions of the array, suggesting that they may subserve different functional roles during behavior, and also suggesting mechanisms by which the rat might uniquely identify the positions and orientations of surfaces around its head. We confirmed key predictions of the functional groupings in a behavioral experiment in which we monitored the contact patterns that the vibrissae made with a flat vertical surface.

## Methods

### Ethics statement

All experimental work was approved in advance by Northwestern University’s Animal Care and Use Committee.

### Modeling whisking kinematics in three dimensions

We used a 3D model of the rat head and vibrissal array (Towal et al., 2011) [7], combined with a kinematic model (Knutsen et al., 2008) [6], to simulate the contact patterns that the vibrissae will make with a planar surface while varying the orientation and distance of the head. Six female Sprague-Dawley rats (*Rattus norvegicus*) were used in the creation of the model and the variation among those individuals is given in Towal et al (2011). The lengths of the whiskers are provided in Table 1. As is standard in the field, the “Greek vibrissae” are defined as the caudal-most vibrissae of rows A-D. These vibrissae are slightly offset in the dorsal-ventral direction from the remaining whiskers in their rows.

**Table 1:** Vibrissal lengths.

*(in mm)*	Greek	Col 1	Col 2	Col 3	Col 4	Col 5	Col 6
**A Row**	46.40	38.47	30.55	22.61	14.68		
**B Row**	48.62	40.69	32.76	24.83	16.90	8.97	
**C Row**	50.85	42.92	34.98	27.05	19.12	11.19	3.26
**D Row**	53.07	45.14	37.21	29.28	21.35	13.41	5.48
**E Row**		47.36	39.43	31.50	23.57	15.64	7.70

The lengths of the vibrissae used in the model [7] are given in mm.

**Fig 1:**
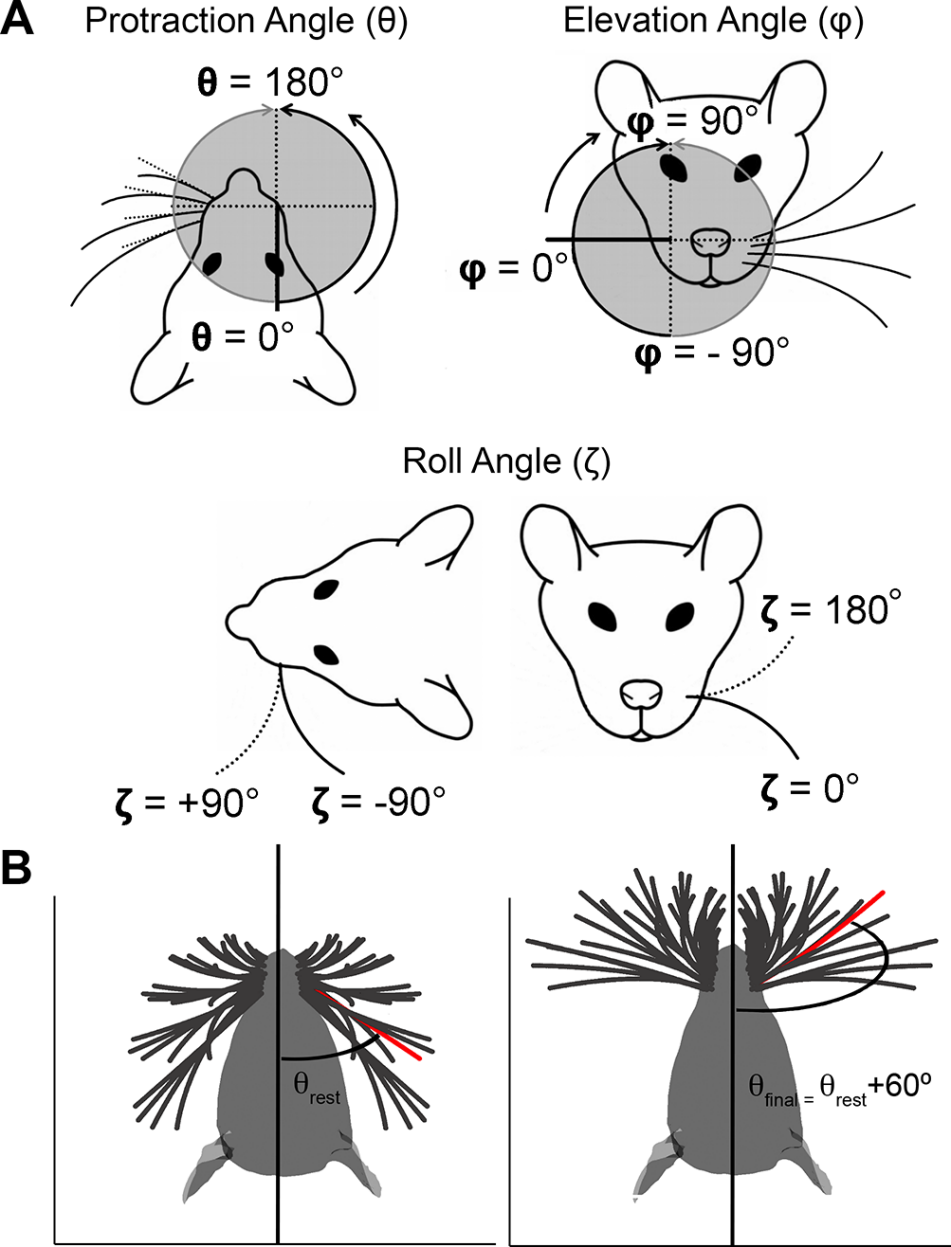
Definitions of whisker angles. **A.** The angle θ is the protraction angle, ϕ is the elevation angle, and ζ is the roll of the vibrissa about its own axis. Figure adapted from Towal et al., 2011 [7]. **B.** With the exception of Fig 5D and Figs 15–17, all simulation results were obtained by protracting the vibrissae 60° from biomechanical rest. A 60° protraction from rest brings the rostral-most whiskers to an angular position of 172° and the caudal-most (Greek) whiskers to 108°. The C1 vibrissa is shown in red for reference.

Fig 1 illustrates the variables used to describe the position and orientation of a vibrissa over the course of a whisk. The variables are the protaction angle θ, the elevation angle ϕ, and the roll of the vibrissa about its own axis, ζ.

Towal et al (2011) provides the value of the resting angles θ_0_ and ζ_0_ (and their associated error bounds) for each vibrissa in the array. Knutsen et al (2008) provides values for the resting angle ϕ_0_ and its associated error bounds.

The Knutsen study (2008) also identifies the slopes of the relationships between θ and ϕ, and between θ and ζ during whisking motion in the awake, behaving animal. Based on these slopes, we can write equations that describe how the vibrissae will roll and elevate as a function of protraction angle, θ. These kinematic equations are shown in Table 2.

**Table 2:** Equations for vibrissal kinematics.

Row	Equation for θ	Equation for ϕ	Equation for ζ
A	θ = 0.1° /timestep	ϕ = (56 ± 5.3) + 0.12•dθ	ζ = ζ_0_ - (0.76 ± 0.08)•dθ
B	θ = 0.1° /timestep	ϕ = (25 ± 9.4)+ 0.30•dθ	ζ = ζ_0_ - (0.25 ± 0.18)•dθ
C	θ = 0.1° /timestep	ϕ = (-4.2 ± 6.3) + 0.30•dθ	ζ = ζ_0_ + (0.22 ± 0.22)•dθ
D	θ = 0.1° /timestep	ϕ = (-27.2 ± 7.7) + 0.14•dθ	ζ = ζ_0_ + (0.43 ± 0.11)•dθ
E	θ = 0.1° /timestep	ϕ = (-44 ± 7.6) + 0.02•dθ	ζ = ζ_0_ + (0.73 ± 0.14)•dθ

Whisker angles ϕ and ζ are functions of the protraction angle θ and the (row, column) identity of the whisker. Numerical values in these equations were obtained from Knutsen et al. (2008) [6]. The resting angle ζ_0_ is unique for each vibrissa and was obtained from Towal et al (2011) [7]. Plus-minus values for ϕ and ζ are error bounds from Knutsen et al. (2008) and are used in the sensitivity analysis of the present study.

The full forward kinematics of 31 vibrissae on the right mystacial pad were simulated by stepping the protraction angle θ in 0.1° increments through a maximum forward protraction of 60° from rest. Data were then reflected about the midline to obtain the trajectories for the left vibrissal array. These angular values bracket the full range of protraction amplitudes typically observed in experimental studies [10–15], although we note that Hill et al. 2008 occasionally observed protraction amplitudes up to 65°. Fig 1B illustrates the 60° protraction angle used in the present study, providing an intuition for the angles spanned.

Note that the starting position for all protractions was biomechanical rest, i.e., the angular position at which none of the facial muscles are contracted. A 60° protraction from rest brings the rostral-most whiskers to an angular position of 172° and the caudal-most (Greek) whiskers to 108°. Previous studies that cite protraction amplitudes of 100° or even 110° fall within this range [11,12]. These previous studies report such large amplitudes for two reasons. First, the studies measure protraction from the “start” of a whisk, where “start” is an angular position retracted relative to biomechanical rest. Second, these studies used an optical sensor that the authors state is likely to have overestimated the amplitude of the whisk [11,12].

In a subset of analyses used to assess the location of the “blind-spot” (Results: “Quantifying the ‘unreachable space’…”), we increased protraction amplitude up to 90°, beyond a physically realistic range. The goal of extending this analysis to the non-physical domain was to control for the possibility that the blind spot was an artifact of insufficient protraction. Non-physical configurations, in which the whiskers began to cross over each other and penetrate the rat’s head emerged for some vibrissa at protraction angles even as small as 70°.

In an additional set of analyses (Results: “Extreme Whisking…") we examined the mappings with the assumption that the whiskers start from positions retracted all the way back against the head, and protract to extreme values (up to 182° for the rostral-most whiskers and a minimum of 148° for the Greek column).

We emphasize that the equations used to simulate whisking kinematics were obtained from a study performed in awake behaving rats [6]. The kinematic equations of the present study thus inherently include the effects of both extrinsic (mystacial pad) muscles as well as intrinsic (sling) muscles. The simulations include the changes in elevation and roll observed in awake animals [6,16–18]. Additionally, because the results depend only on the geometric angle of first contact with the surface, inter-whisker effects such as spread [19,20] will not affect the results.

### Head position and orientation relative to the planar surface

**Fig 2:**
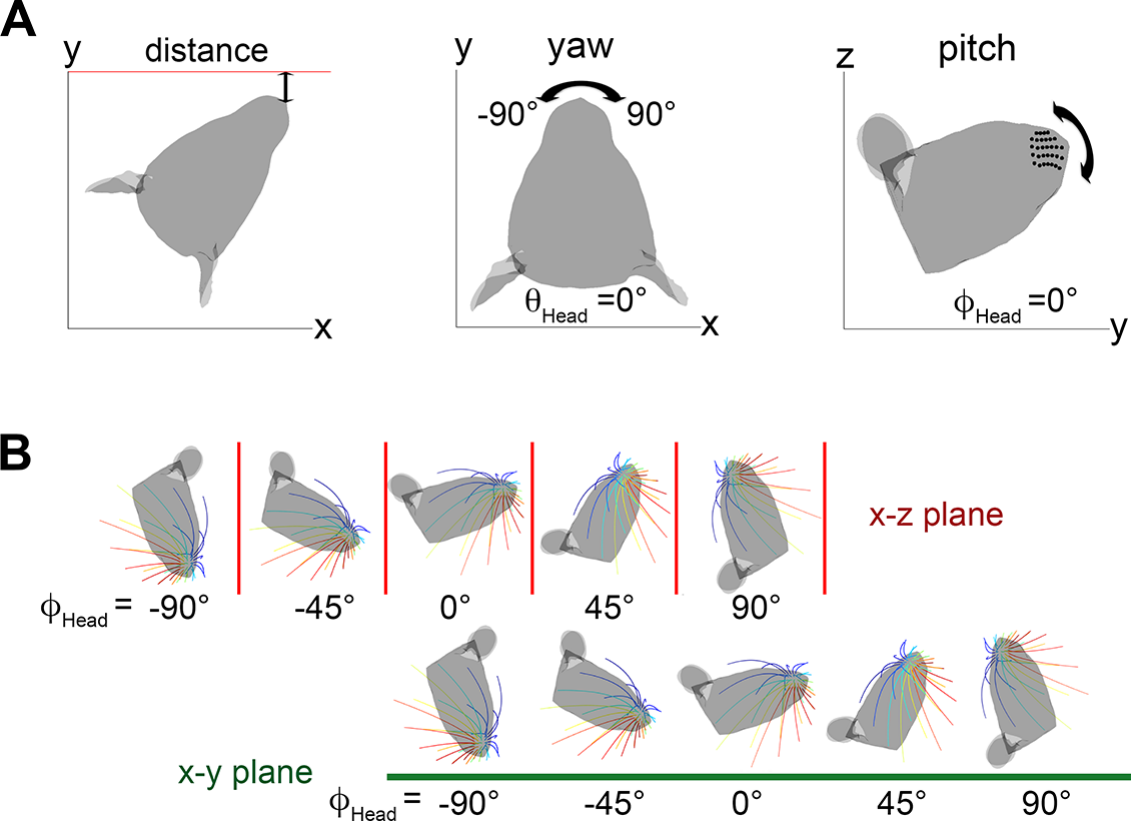
Definitions of head angles. **A.** Distance, yaw, and pitch are defined for the head. Following the convention of Towal et al. (2011), a head pitch of ϕ_head_ = 0° was defined as the angle at which the whisker rows are parallel to the ground. In this orientation the rat’s head is tilted slightly upward [7]. **B.** In simulation, the pitch of the head was varied from ϕ_head_ = -90° to ϕ_head_ = +90°. The top row shows these orientations relative to a vertical wall. The bottom row shows the same head pitches relative to the ground (an offset of -90°).

To simulate a rat exploring a flat surface, a vertical wall was represented as an infinite x-z plane at different distances from the rat’s nose. At each distance, the head was rotated through different angles to simulate different relative angles between the head and the wall. Fig 2A illustrates the conventions used to describe the distance of the head to the wall, and the yaw and pitch of the rat’s head.

The “*distance”* (d) is measured between the midpoint of the nostrils and the wall.

The “*yaw”* of the head (θ_head_) is defined as zero when the rostrocaudal midline of the head is in the yz-plane and the caudal-to-rostral vector points in the positive y-direction. Values of θ_head_ between -90 and 0 correspond to the right vibrissae turned toward the wall while values between 0 and 90 correspond to the left whiskers turned toward the wall.

The “*pitch”* of the head (ϕ_head_ is defined to be zero when the average plane of the whisker rows lies parallel to the xy-plane. Note that this means that ϕ_head_ = 0° corresponds to the rat looking slightly upward, and ϕ_head_ = -20° corresponds to the head being approximately level with the ground. Informal observations from our laboratory and others indicate that typical head pitches during locomotion are roughly between -25° and -45°.

In simulations, the distance of the rat’s nose to the wall was varied from 0 to 60 mm in 1 mm increments along the y-axis and both the head yaw and pitch were varied from -90° to +90° in 5° increments. As shown in Table 3, running the simulation over the entire range of (d, θ_head_, ϕ_head_) resulted in a total of 83,509 configurations.

**Table 3:** Head positions and orientations relative to a vertical surface.

*Variable*	*Min*	*Max*	*Increment*	*Number of values tested*
Distance from nose to wall (d)	0 mm	60 mm	1 mm	61
Head Yaw (θ_head_)	-90°	90°	5°	37
Head Pitch (ϕ _head_)	-90°	90°	5°	37
Total number of configurations = 61*37*37				**83,509**

The range of head pitches, yaws, and distances over which the simulation was run results in 83,509 configurations.

Note that a head pitch near ϕ_head_ = 90° is unnatural for exploring a vertical surface. However, these pitches are equivalent to simulating vibrissal contacts with a horizontal surface (i.e., the ground). The equivalency between these orientations is shown in Fig 2B.

### Definitions of θ_impact_, resting-contacts, no-contacts, and whisking-contacts

**Fig 3:**
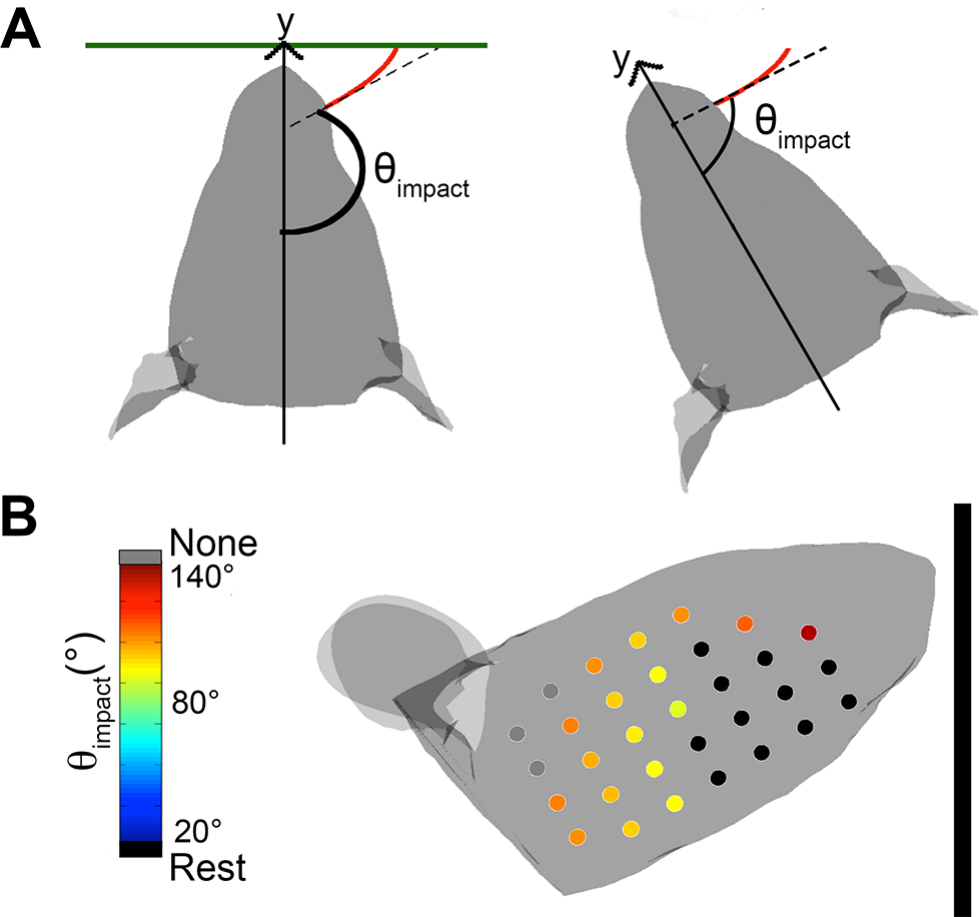
Definitions of contact angles during whisking behavior. **A.** θ_impact_ is defined as the angle between the rostral-caudal axis and the vector tangent to the base of the vibrissa when it first makes contact with the object. Note that the illustrated curvature of the whiskers reflects their intrinsic curvature and does not simulate the whisker bending against the surface. **B.** Color code for the different types of vibrissal-object contact. Vibrissae in contact with the surface before protraction begins (resting-contacts) are plotted as black circles on a schematic of the mystacial pad. Vibrissae that never contact the surface at any point during the whisk (“no-contacts”) are shown in gray. Vibrissae that make contact at a particular protraction value of the whisk are color coded according to θ_impact_.

During navigation and exploration rats must localize objects in head-centered coordinates. In head-centered coordinates, the angle at which a vibrissa initially touches an object is defined by the variable θ_impact_ [7–9]. As shown in Fig 3A, θ_impact_ is defined as the angle between the rostrocaudal midline of the rat and the vector tangent to the vibrissal base when the vibrissa first impacts the object. Previous studies have shown that the rat can determine θ_impact_ to high precision [8,9], and it is therefore the parameter quantified in the present study.

Each vibrissa was simulated to have 100 nodes. Because the whiskers have a significant intrinsic curvature, and because the whiskers roll and elevate during a whisk, it is not at all guaranteed that a whisker will always make contact with the surface at its tip. A vibrissa was determined to have collided with the planar surface when the coordinates of any of its nodes crossed the boundary of the surface.

For each head configuration relative to the wall (d, θ_head_, ϕ_head_ as listed in Table 3), a vibrissal protraction of 60° was simulated. Each time that a vibrissa-object collision occurred the whisker’s (row, column) identity, the node of contact, and θ_impact_ were recorded to a MySQL database. Given that the goal of this study was to determine relative surface orientation based on geometric parameters alone (θ_impact_), whisker bending was not simulated and no data are reported on whisker angles after the first contact.

Fig 3B illustrates three types of contacts that occurred in simulation. Depending on the orientation of the head and its distance from the surface, some vibrissae may be in contact with the surface when they were at rest, even before protraction begins. These contacts have been termed “*resting-contacts*,” and throughout the paper they are color-coded in black. Other vibrissae may never contact the surface at any point during the full whisk. These have been termed “*no-contacts*,” and are color-coded in gray or white. Finally, some vibrissae are not in contact with the surface when protraction starts, but come into contact with the surface after a certain amount of protraction. These have been termed “*whisking-contacts*.” Throughout the paper, whisking-contacts are color-coded to indicate the value of θ_impact_.

### Sensitivity analysis and consideration of non-flat surfaces

We performed a sensitivity analysis to demonstrate that the results presented here were robust to uncertainty in model parameters and thus to differences between individual rats. We allowed six parameters to vary independently and examined their effect on the values of θ_impact_:

- Whisker lengths were allowed to be shorter than the nominal by up to 20%. The percent deviation from nominal was uniformly distributed over this range.
- Values for the resting angles ϕ_0_ and ζ_0_ were uniformly distributed between ±20% of their nominal value.
- Values for ϕ and **ζ** in the kinematic equations were normally distributed between the error bounds listed in Table 2.
- Basepoint location was allowed to vary by up to 15% of the distance between neighboring basepoints and uniformly in any direction.

The sensitivity analysis was run over a representative range of configurations: distances between 0 and 60mm in 5mm increments, pitch between -90° and 90° in 30 degree increments, and yaw held constant at 0°. This yielded a total of 42 configurations. These configurations were chosen because they put the greatest number and variety of vibrissae in contact with the wall. Because error magnitude was found to be essentially random across configurations, the analysis was not extended to higher resolution or to include all of the different yaws. For each of the 42 configurations, 1,000 different simulations were run in which the six parameters were allowed to vary.

**Fig 4:**
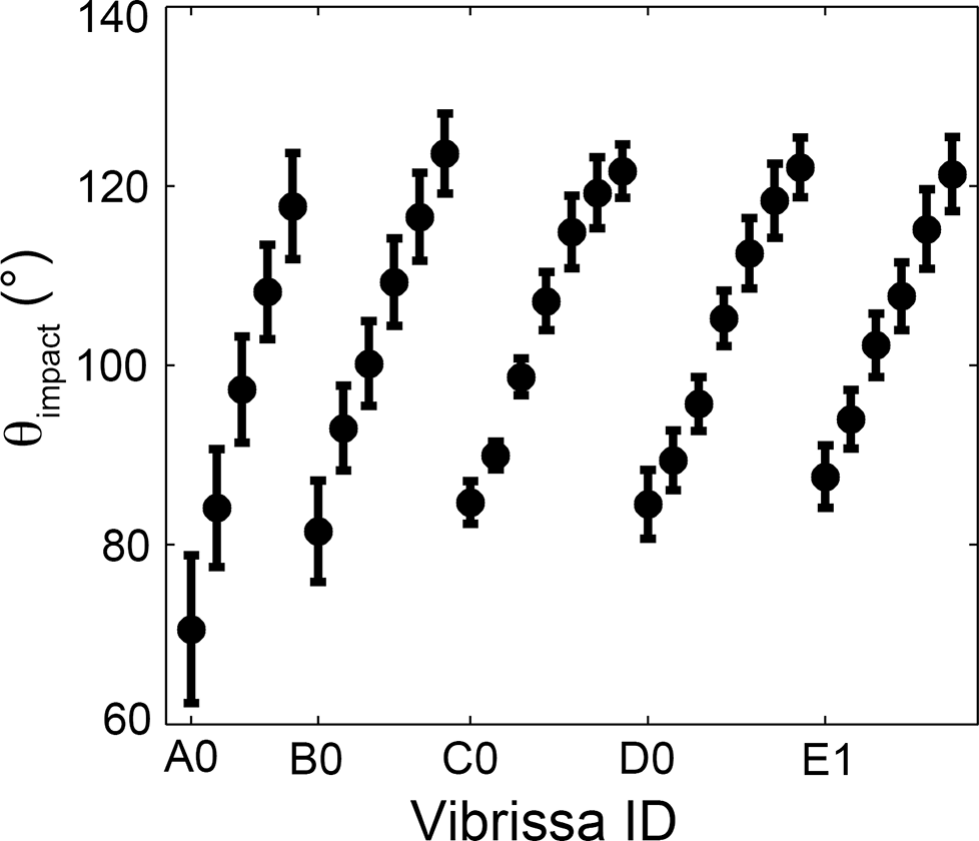
Effect of parameter variations on θ_impact_. The mean and standard deviation of θ_impact_ for each vibrissa across 42,000 parameter-varied simulations is plotted.

In this manner, we obtained values for θ_impact_ over a total of 42,000 parameter-varied simulations. Fig 4 shows the average value of θ_impact_ for each of the vibrissae across the 42,000 trials. Errorbars show the standard deviation around the mean. Most vibrissae have a standard deviation of less than 10°, and adjacent vibrissae contain distinct ranges of θ_impact_.

Although the present simulations describe vibrissal-object contact patterns for a flat surface only, we also considered the possibility that the surface was not perfectly flat. We found that as the radius of curvature was decreased from infinite (perfectly flat) to 140mm (5.5 inches) the change in θ_impact_ was less than 0.1deg/mm. Thus the general results of the present work do not rely on the surface being perfectly flat. An extremely conservative estimate is that results will hold as the rat encounters any surface that is no more curved than, say, a large dinner plate (~5.5 inch radius of curvature, 11 inch diameter). Patterns of vibrissal-object contact for surfaces with greater curvature will be the topic of a future study.

### Measurement of head orientation and whisker contacts during exploration

We compared simulation results with results obtained in an experiment in which rats were allowed to freely explore a flat, vertical wall. In this experiment, five female Long-Evans rats (*Rattus norvegicus*) perched on a ledge and freely explored a wall placed on the other side of a gap. The wall was made of glass, had no textural features, and was large enough so that rats could not touch the edges of the wall. All rats were naive to the task and all experiments were performed under infrared (IR) lighting.

Two high-speed video cameras (1,000 frames per second, Photron, San Diego, CA) recorded the rats as they explored the wall. One camera captured a “bird’s-eye” view and the second a “head-on” view through the glass wall. Video recording was triggered by an IR sensor when the rat approached the glass and recording continued until the end of the trial.

To reconstruct 3D head position and orientation, we tracked each rat’s eyes (semi-automatically) and nose (manually) in both camera views. The trajectories of the eyes and nose were filtered at 20 Hz, and the two-dimensional (2D) trajectories were then merged into 3D using standard image processing techniques [21].

Methods to detect and quantify the locations of vibrissal-object contact have been described in detail previously [22]: a beam from a near-infrared (975 nm) laser diode was passed through a series of optics to produce a 2mm-thick collimated plane of light directly in front of the glass sheet. As the rat whisked into the glass sheet, the whiskers broke the plane of the light sheet, scattering light at the point of contact. These points of light were captured by the high-speed video cameras. Independent image calibration determined what light intensity corresponded to the whiskers making contact with the glass; intensities below this threshold were determined to have been within the light sheet but not in contact with the glass, and were discarded. Contact points were identified and tracked using image processing algorithms based on particle tracking velocimetry.

## Results

Throughout *Results*, the parameters θ and ϕ refer to θ_head_ and ϕ_head_, the yaw and pitch of the rat’s head relative to a flat surface. The horizontal angle at which each vibrissa first touches the surface is called θ_impact_.

It is important to remember that throughout the present study the head pitch and yaw θ and ϕ refer to the *relative* angles between the rat’s head and the surface. During natural exploration, the rat might encounter a surface at any possible orientation relative to its head. The present study does *not* suggest that rats use tactile inputs from their vibrissae to determine the orientation of their head relative to their body or relative to gravity; vestibular and proprioceptive cues from the neck muscles could serve this purpose [23]. We also note that the present work focuses entirely on contact angle as a cue for surface position and orientation. Timing information may also be used as a cue but falls outside of the scope of the present work.

We first identify the remarkably small number of head configurations that result in zero vibrissae touching the surface. We then build off previous studies that have shown that rats can determine θ_impact_ with very high resolution [9,24]. We aimed to identify the relationship between θ_impact_ across the vibrissae in the array, and the distance, pitch and yaw of the surface relative to the rat’s head.

### Quantifying the “unreachable space” and the “blind spot” of the vibrissal array

We varied distance, pitch, and yaw to determine the “reachable configuration space” for the vibrissal array. For the purposes of the present paper we define the reachable configuration space as the head poses in which at least one whisker is guaranteed to make contact with the surface over the course of a simulated whisk.

**Fig 5:**
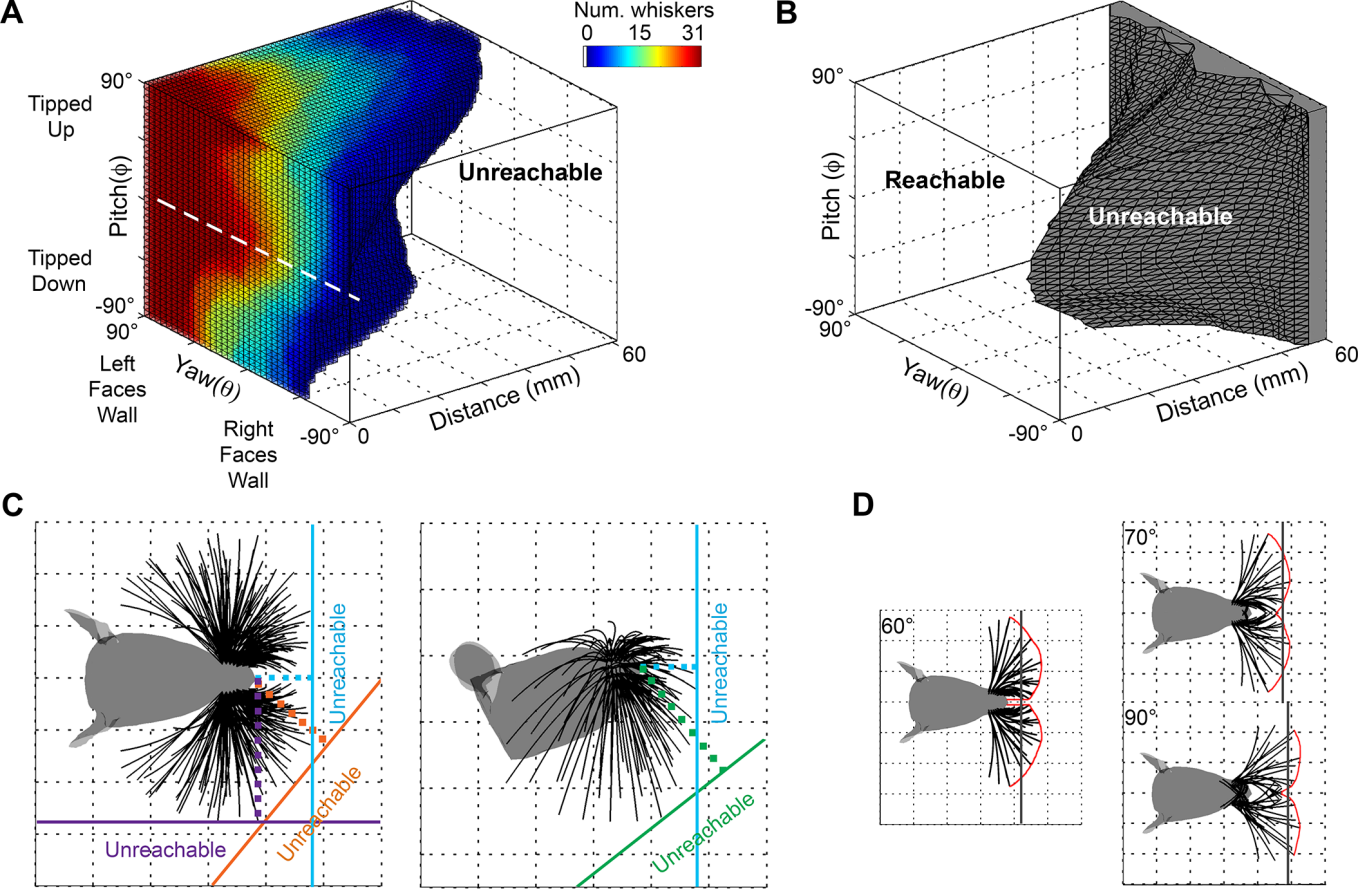
Reachable configuration space of the array. **A.** Reachable space for the 31 vibrissae of the left array. The figure shows the configurations that allow at least one vibrissa of the left array to come in contact with an infinite surface. Both resting-contacts and whisking-contacts are included. Color indicates the number of vibrissae that make contact. Configurations in which no vibrissae contact the surface are left white (labeled “unreachable”). The broad red peak at the level of the white dashed line indicates that at any given value of yaw, pitches around 0° will maximize the number of whisker contacts with the surface. **B.** Reachable and unreachable spaces for both sides of the array acting together. The configuration space over which no vibrissae, from either the right or left side of the array, make contact is extremely small until the distance becomes large. The unreachable region is colored gray and labeled “unreachable.” **C.** Example “walls” at different orientations relative to the rat’s head demarcate the unreachable space. The dotted lines show that a wall directly in front of the rat’s nose (pitch = yaw = 0°, cyan) becomes unreachable at a smaller distance than a wall to the rat’s side (orange and purple walls) or below the rat (green wall). **D.** The “blind spot” is very different from the unreachable space. The blind spot is a small (x, y, z) region of space that the whiskers cannot touch, even at extreme protraction angles. The left subplot indicates a protraction of 60° from rest, and the blind spot takes the form of a niche in front of the rat’s nose. At protractions of 70° and 90° (right plot), a number of simulated whiskers become un-physical (they penetrate the rat’s face due to excessive protraction) and yet the small blind spot remains. Note that an imaginary vertical surface (gray line) passing through a central point in the blind spot is not un-reachable because some vibrissae can still contact it.

The reachable space for the left side of the array is illustrated in Fig 5A. The number of vibrissae in contact with the surface is shown as a function of distance, pitch, and yaw. The mapping for the right array would appear identical except that the yaw axis would be exactly inverted. The most obvious feature in the figure is that more vibrissae of the left array come in contact with the surface when the rat’s left side faces the wall. The second most noticeable feature is the broad peak centered around 0° pitch, indicated with a white dashed line. This peak indicates that at any given value of yaw, pitches around 0° (with the rat’s head approximately level) will maximize the number of whisker contacts with the surface.

Fig 5A illustrates the reachable configuration space for the left side of the array only, but when both sides of the array work in concert, the reachable space grows dramatically. Fig 5B illustrates the regions with no contacts from either side of the array. This plot is now symmetric in yaw, because as one side turns towards the wall the other turns away.

The “unreachable space” can be thought of as the distance at which an infinite vertical wall “disappears,” and it is a function of the pitch and yaw of the rat’s head. It answers the question: given a specific pitch-yaw configuration, at what distance will an infinite wall no longer be detectable by any vibrissae? To give further intuition for this definition, four “example walls” at different orientations relative to the rat’s head are shown in Fig 5C. Each example wall is shown in a different color, and the unreachable space is labeled in the same color. The surprising result is that the reachable distance is smallest for orientations near pitch = 0° and yaw = 0°, corresponding to the rat directly facing the wall. In contrast, the rat can sense a wall to its side (purple and orange “walls”) or below it (green “wall”) at a much further distance than it can a wall directly in front of its nose (cyan “wall”).

With this interpretation in mind, we return to the characterization of this unreachable space. Fig 5B shows that when the head is within 16 mm of the surface, all configurations are guaranteed to have at least one vibrissa in contact. Within 25 mm of the wall only 1.5% of configurations (529 out of 35,594) are unreachable by any vibrissae. As the distance increases to 40 mm, this percentage grows to 8.1% (4547 out of 56,129), and by 60 mm approximately 25.7% (21,455 out of 83,509) are unreachable. Again, the center of this unreachable space corresponds to pitches and yaws near 0°, meaning that rat has the smallest preview distance right in front of its nose.

In summary, Fig 5A–5C show that when the rat faces an object with right-left symmetry and an approximately level head, the reachable distance is the smallest, but within that distance the number of whiskers in contact with the surface is maximized.

The region of unreachable space depicted in Fig 5A–5C implicitly invokes the idea of a “blind spot.” In contrast to the unreachable space, the “blind spot” can be thought of as the set of (x, y, z) points in space which cannot, even with an extreme degree of protraction, be reached by any vibrissae. The “blind spot” is defined entirely in head-centered coordinates, and is completely determined by the morphology of the array and its sensory volume [20].

For a protraction of 60°, the blind spot takes the form of a narrow niche directly in front of the rat’s nose, as illustrated in the left subplot of Fig 5D. After a protraction of 70° (top right subplot of Fig 5D) the niche has narrowed to a small cusp. Note that a protraction of 70° results in angular positions greater than 180° for the rostral-most whiskers. A 70° protraction from rest is 5° larger than any experimentally-measured protraction reported for the whiskers of columns 2–6 [10–15]. Also note that the simulated whiskers in this figure begin to unnaturally cross each other. As protraction is increased even further to an even more unnatural 90° (bottom right subplot of Fig 5D) the blind spot remains as a small cusp directly in front of the rat’s nose. Protracting the caudal-most whiskers through 100° does not eliminate the blind spot; this result is not shown in Fig 5, but can be seen later in *Results*: *extreme whisking*.

The intent of this analysis, including such large and unnatural protraction angles, was to ensure that the simulations encompassed the full range of foveal whisking [25]. The blind spot is quite small, but it does exist, even at very high protraction angles. From a behavioral standpoint any head movement is likely to obviate the blind spot, however, it is an inherent geometric feature of the array. Also note that although the specific (x, y, z) regions that define the blind spot are unreachable by any vibrissa, a vertical plane (shown in Fig 5D as a gray line) passing through the central portion of blind spot would be reachable, because several vibrissae are able to make contact at this distance and orientation (illustrated here with pitch = 0°, yaw = 0°).

### Mappings between distance, yaw, and θ_impact_

**Fig 6:**
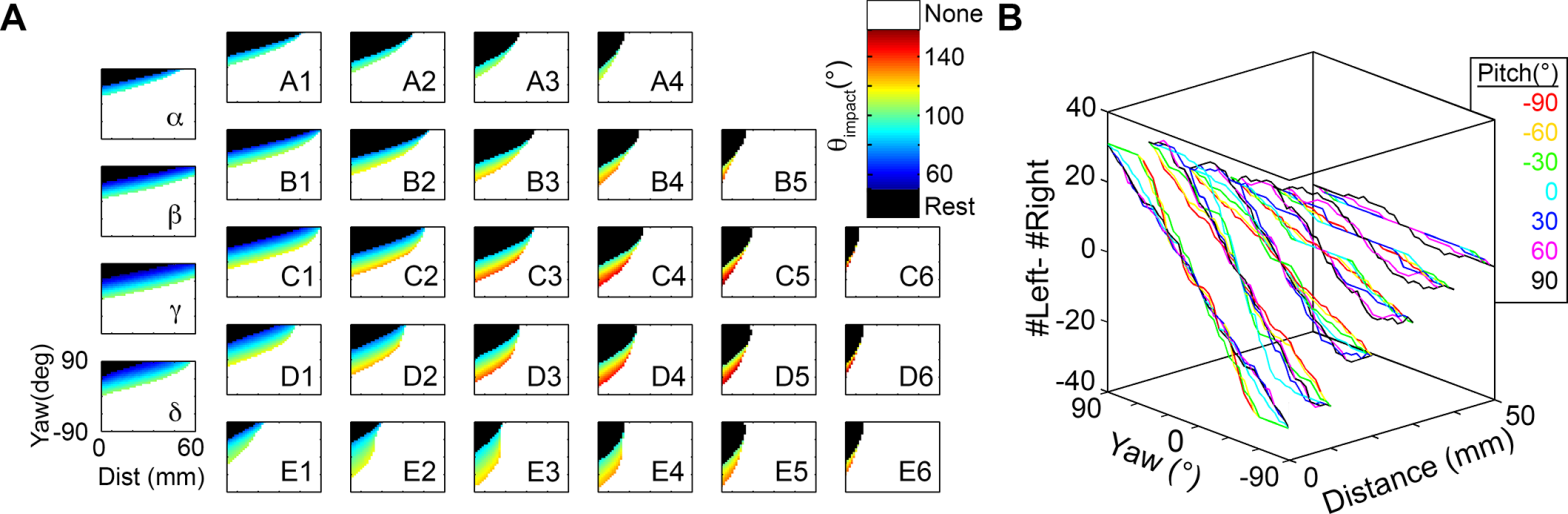
Mappings between distance, yaw, and θ_impact_. **A.** Values of θ_impact_ mapped to yaw and distance for the 31 vibrissae of the left array at pitch = 0°. Resting-contacts are shown in black, while whisking-contacts are color coded according to θ_impact_. A yaw value of +90 means that the left whiskers were turned towards the wall, resulting in a large number of resting-contacts. A yaw value of -90 means that the left whiskers were turned away from the wall, resulting in no contacts (white). **B.** The difference between the number of left and right vibrissae (nL—nR) in contact with the surface is shown as a function of yaw and distance. Each color represents a different pitch value. This difference is seen to increase as the yaw increases, providing the rat with information about yaw.

Having identified the configurations for which one or more whiskers will make contact with the surface, we next focused on quantifying the angle at which each whisker made contact with the surface (θ_impact_), across all different possible distances, yaws, and pitches. Fig 6A illustrates θ_impact_ as a function of yaw and distance relative to the wall for each of the 31 vibrissae on the left side of the array, for the case that pitch = 0° (head tipped slightly upward).

Three features are apparent in Fig 6A. First, the range of yaws over which contact occurs tends to increase from caudal to rostral and from dorsal to ventral across the array. This trend can be observed as an increase in the vertical thickness of the bands in sequential subplots within a row (left to right) and within a column (downwards). Second, the maximum reachable distance increases from rostral to caudal in the array, scaling with vibrissal arc length. This maximum reach occurs for high values of yaw, when the vibrissae are oriented toward the surface. Thus at a pitch near 0°, the rat is able to detect a surface at the greatest distance when the surface is off to its side, with its caudal vibrissae.

The third and most notable feature of Fig 6A, however, is that all vibrissae generally show very similar relationships between yaw, distance, and θ_impact_. For example, all vibrissae exhibit a large number of resting-contacts when one side of the array is oriented directly toward the wall. These resting-contacts are seen as the large black regions in the upper left corners of each subplot. As the rat turns its head to face the wall with more right-left symmetry (yaw = 0°), these resting-contacts give way to whisking-contacts (the colored bands below the black regions in each subplot).

Because these mappings are so similar across vibrissae, it would likely be difficult for the rat to extract any information about the yaw of its head relative to the surface by comparing θ_impact_ across vibrissae on the same side of the array. Instead, it seems most likely that information about yaw comes from comparisons between the right and left arrays, as shown in Fig 6B. The difference between the number of left and right vibrissae (nL—nR) in contact with the surface is a function of yaw and distance, but relatively independent of pitch. At any given distance, the difference between the number of left and right vibrissae in contact increases approximately linearly with yaw. The pitch of the rat’s head, represented by different colors, does not have a strong effect on this linear relationship.

The results of Fig 6B have several implications. First, even if the rat has no estimate of the distance to or pitch of the surface, the value of nL—nR will provide a general estimate of yaw: nL—nR = 0 corresponds to a yaw near 0° while a greater difference between nL and nR signifies a larger absolute yaw. Second, knowledge of both distance and nL-nR is sufficient to determine yaw, but knowledge of yaw and nL-nR is not necessarily sufficient to determine distance (e.g., there are multiple distance values for nL-nR = 0, at zero yaw). Finally, the slope of the relationship between yaw and nL-nR increases as the distance decreases, i.e. as the rat moves closer to the surface. The rat could therefore use this change in slope across two whisks to determine information about both yaw and distance.

### Each whisker has a characteristic mapping between distance, pitch, and θ_impact_

**Fig 7:**
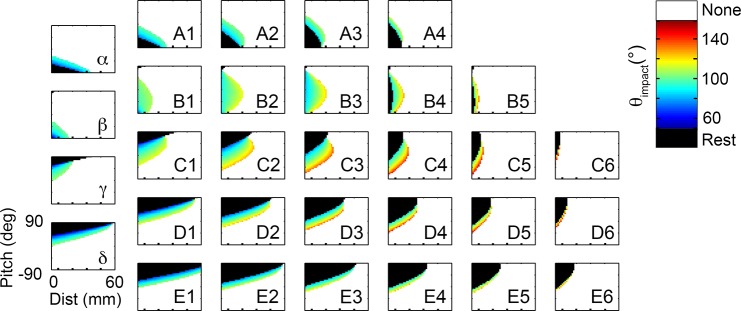
Mappings between distance, pitch, and θ_impact_ for the 31 vibrissae of the left array. Resting-contacts are shown in black, while whisking-contacts are color coded according to θ_impact_. A pitch value of +90 means that the head is pitched up and a pitch value of -90 means that the head is pitched down. Yaw is constant at 0°.

In contrast to the relatively consistent results for yaw described above (Fig 6A), each vibrissa was found to have a characteristic mapping between θ_impact_ and the pitch-distance configuration. This result is shown in Fig 7, which plots θ_impact_ as a function of head pitch and distance for each of the 31 vibrissae of the left side of the array, for the case that yaw = 0°. The figure reveals that many parameters change from vibrissa to vibrissa, including the ratio of resting-contacts to whisking-contacts, the range of distances and head-pitches over which contact can occur, and the overall shape of the plot.

In particular, the proportion of resting-contacts to whisking-contacts varies dramatically across vibrissae. The interior B and C rows show mappings that include a large number of whisking-contacts (colors), while the exterior rows, A, D and E, are dominated by resting-contacts (black).

Further inspection of Fig 7 hints at characteristic groupings of vibrissae: The dorsal A row is very nearly a mirror image of the ventral D and E rows, and these vibrissae reach their maximum distance at large pitch magnitudes. In contrast, vibrissae of the interior B and C rows reach their maximum distance at small pitch magnitudes. These groupings suggest specific functional roles for different groups of vibrissae; the next sections explore these characteristic groups in greater detail.

### The exterior rows of vibrissae (rows A, D, and E) provide key information about relative pitch

**Fig 8:**
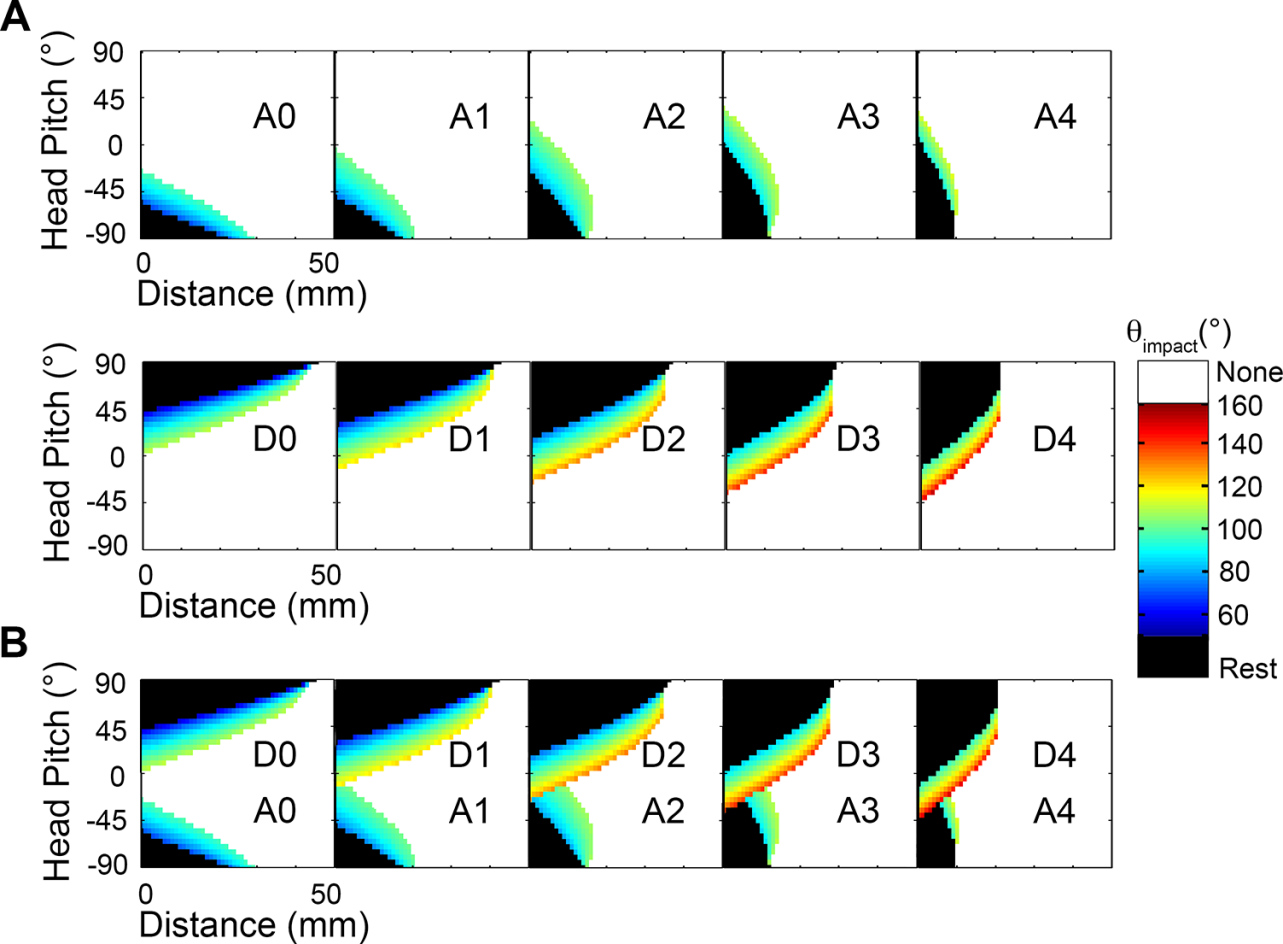
Vibrissae in exterior rows constrain values of relative pitch. **A**. Vibrissae of the A-row show a relationship between θ_impact_, pitch, and distance that are close to a mirror image of the mappings for the D and E row vibrissae. Mappings for the E row are not shown, but Fig 7 shows they will closely resemble the mappings of the D row. **B.** Overlaying the mappings for dorsal and ventral vibrissae constrains the possible values of head pitch. The mappings of both **(A)** and **(B)** are shown for a value of yaw = 0°, but similar results were found for all values of yaw. As in all previous figures, resting-contacts are shown in black, while whisking-contacts are color coded according to θ_impact_.

We begin our detailed investigation of the functional groupings suggested in Fig 7 by considering the exterior rows (A, D, and E). The vibrissae of the A and D rows generate contact patterns that resemble mirror images of each other, as shown in the mappings of Fig 8. The large black regions in each subplot indicate that the contact patterns are dominated by resting-contacts for moderate to large head pitches. Whisking-contacts occur only over a small range of head-pitches, as indicated by the narrow colored regions.

The A-row vibrissae are dominated by resting-contacts when the head is pitched downwards, and by whisking-contacts as the head becomes more level. The D- and E-rows show exactly the opposite trend: resting contacts tend to occur when the head is pitched up, while whisking-contacts characterize head pitches near zero. These high pitch angles are equivalent to ground contact (c.f., Fig 2), suggesting a particularly important role for the ventral vibrissae in detecting and exploring variations in the ground surface.

Because the dorsal and ventral rows exhibit opposite trends, overlapping their contact patterns constrains the possible values of head pitch, as shown in Fig 8B. Resting contacts by the vibrissae of the D and E rows will indicate to the rat that its head is pitched upwards relative to the surface. Resting contacts by the A row suggest negative (downward) head pitches. At intermediate pitch values, the value of θ_impact_ for each whisker provides additional information about both pitch and distance. However, only the rostral-most vibrissae of the exterior rows are able to make contact with the wall at a level pitch, and those only at very small distances.

Summarizing so far, comparing contact patterns across the exterior rows of vibrissae could provide the rat with significant information about pitch. We next asked what information the exterior rows might provide about distance to the surface. The diagonal bands of whisking contacts shown in Fig 8 show that θ_impact_ is a function of both distance and pitch. Consider, for example, the D2 vibrissa: a vertical line drawn at a distance of 20mm shows that θ_impact_ changes from resting contacts (black) to whisking contacts (colored) around a pitch of 45°. θ_impact_ then increases (progresses from blue to orange) as the pitch continues to decrease. Likewise, a horizontal line drawn at a pitch of 30° shows a similar progression from resting contacts to whisking contacts (at a distance of 10mm), and from low θ_impact_ (blue) to high θ_impact_ (orange), as distance is increased. Thus, although the rat could estimate pitch by comparing contact patterns across the A and D/E rows, the value of θ_impact_ seems unlikely to provide robust cues about distance—the correlation between θ_impact_ and distance will change over a range of pitches. This correlation will be explored further in a subsequent section (“The two interior rows…show a correlation”).

### All whiskers of the B and C rows have their maximal reach for level-head pitches

We continued our investigation of the functional groupings by quantifying the mappings for the interior rows, B and C. These two rows show contact patterns very different from those of the exterior rows described above. An initial analysis of the B- and C- row mappings demonstrated that the patterns of the B-row whiskers are more intuitive than those of the C-row. We therefore first describe key results in terms of the B3 whisker, and then extend the results to the C2 whisker, which has been investigated extensively in many previous physiological studies [26–29].

**Fig 9:**
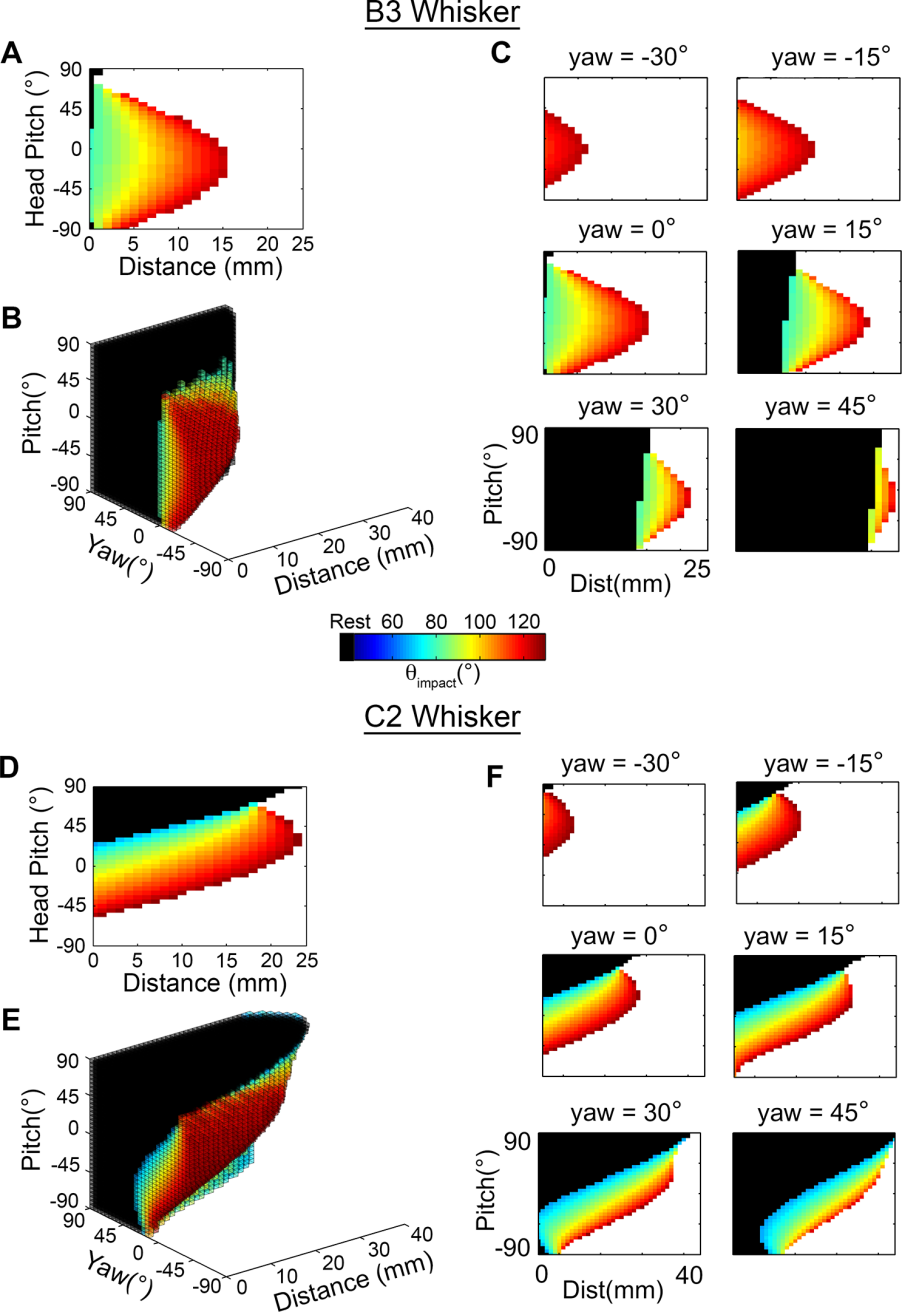
Mappings of θ_impact_ for the left B3 and C2 vibrissae. In all subplots black corresponds to resting contacts, white corresponds to no contacts, and the value of θ_impact_ is represented in the color scale. **A.** The B3 vibrissa is characteristic of the B and C rows, which show a correlation between θ_impact_ and distance. The mapping is shown for yaw = 0°. **B.** The 3D mapping for the B3 vibrissa is convex near pitch and yaw values of 0°. Note that subplot A is a vertical cross section through this larger space. **C.** As the rat turns towards the wall the number of resting-contacts increases. For whisking-contacts, the overall functional relationship between θ_impact_ and distance remains the same as seen in subplot A, regardless of yaw. **D.** The C2 vibrissa shows characteristics of the interior row, although is less symmetric about pitch = 0° than the B3 vibrissa. **E.** The 3D mapping of the C2 vibrissa shows the same convex structure as other vibrissae of the interior rows. **F.** The relationship between distance, pitch, and θ_impact_ is shown for the C2 vibrissa for different values of yaw. Large positive values of yaw are again dominated by resting-contacts.

The mapping for the B3 whisker is shown in Fig 9A, for a yaw of 0°. The most obvious feature is that the mappings for the B3 whisker look exactly like one might “expect” them to look. Several features make the B3 mapping intuitive. First, θ_impact_ scales with distance. Second, the whisker has its farthest reach for a range of near-level pitches (between 0° and -35°), observed as a peak near pitch = -20° in Fig 9A. Third, the rate at which θ_impact_ decreases is symmetric with pitch. The central and symmetric peak of this mapping is so intuitive, that one might have expected all whiskers to show mappings that are simply scaled versions of B3.

Fig 9A shows results for a single value of yaw, but in fact the intuitiveness of the mappings is retained over all yaws, as shown in the complete distance-pitch-yaw mapping of Fig 9B. As expected from the location of the peak in the 2D mapping, the 3D mapping is convex near pitch and yaw values close to zero. This means that an increased number of contacts will tend to occur when the rat’s head is close to level with the ground and faces the surface with right-left symmetry. If the mapping were concave, the rat would have to turn to the side or pitch its head up/down to make additional contact with the surface. All vibrissae of the B and C rows were found to exhibit similar convex outwards mappings, with the exception of the beta and gamma whiskers, which will be discussed later.

The 3D mapping in Fig 9B also highlights that over all configurations exhibiting contact, well over half are resting contacts. The resting contacts tend to dominate larger, positive values of yaw, when the vibrissae are oriented towards the surface. Fig 9C illustrates six horizontal cross-sections through Fig 9B, more clearly showing the increased fraction of resting contacts as the rat’s cheek turns to face the wall. These subplots also confirm that the reach of the B3 vibrissa is maximized for pitches between 0° and -35°, regardless of the value of yaw.

Although this section has described results only for the B3 whisker, similar results were found to hold for all whiskers of the B and C rows. Because the C2 vibrissa is often the focus of electrophysiological studies, the analysis described above for B3 is repeated here for C2 and shown in Fig 8D–8F. The C2 whisker shows features of both the B-row and the D-row. Specifically, it resembles the B3 whisker in having a peak—corresponding to maximum reach—for pitch values near zero. The peak for the C2 whisker occurs between 20° and 30° (Fig 9D). However, the C2 mapping also exhibits some of the elongated-angled structure seen for the D and E rows. Examining the 3D structure (Fig 9E) shows that the C2 mapping is similar to the B3 mapping, but it is “elongated,” in a manner similar to mappings of the D row. Different yaw slices through this three-dimensional space illustrate the dominance of resting contacts for positive yaw values (Fig 9F), and emphasize that at high values of yaw the C2 whisker begins to look more like a D row whisker. Summarizing, the convex structure of the C2 mapping allows for a maximal reach at close-to-level head pitches, but as the head pitches and yaws more, we see a large increase in resting contacts.

### The two interior rows (B and C) of vibrissae show a correlation between θ_impact_ and distance that is robust to changes in pitch

Recall that the first section of results showed that the rat could compare the number of whiskers in contact on the left versus right side of the array to obtain an estimate of yaw. Next, we showed that the rat could estimate pitch by comparing contact patterns across the exterior rows (i.e., A and E or A and D). These results also hinted that the exterior rows might not provide robust cues about distance, because the angled structure of the mappings clearly showed that θ_impact_ depended on both distance and pitch.

In contrast to the exterior rows, the relatively symmetric (in pitch) mappings associated with the B and C rows (Fig 9) might now lead one to wonder whether θ_impact_ for these rows would provide the rat with robust distance cues. For example, in Fig 9A it is clear that a given value of θ_impact_ does not correspond to a unique value of distance, but it does tightly constrain the possible range of distances—a value of θ_impact_ = ~95° indicates that the rat’s snout is between 0 and 6 mm of the surface.

Intuitively, θ_impact_ is expected to provide the rat with information about the distance to the surface: if a greater protraction is required for contact, the surface must be further away. Consistent with this intuition, quantitative analysis showed that θ_impact_ does have a strong correlation with distance, but only if pitch and yaw are known exactly. Specifically, for a given pitch and yaw, every whisker shows an extremely strong correlation between distance and θ_impact_ (above r = 0.99 for all whiskers).

But what if yaw and pitch are not exactly known? The orientation of the head greatly alters the relationship between θ_impact_ and distance. We examined how much variations in yaw and pitch influenced the robustness of the correlation between θ_impact_ and distance.

**Fig 10:**
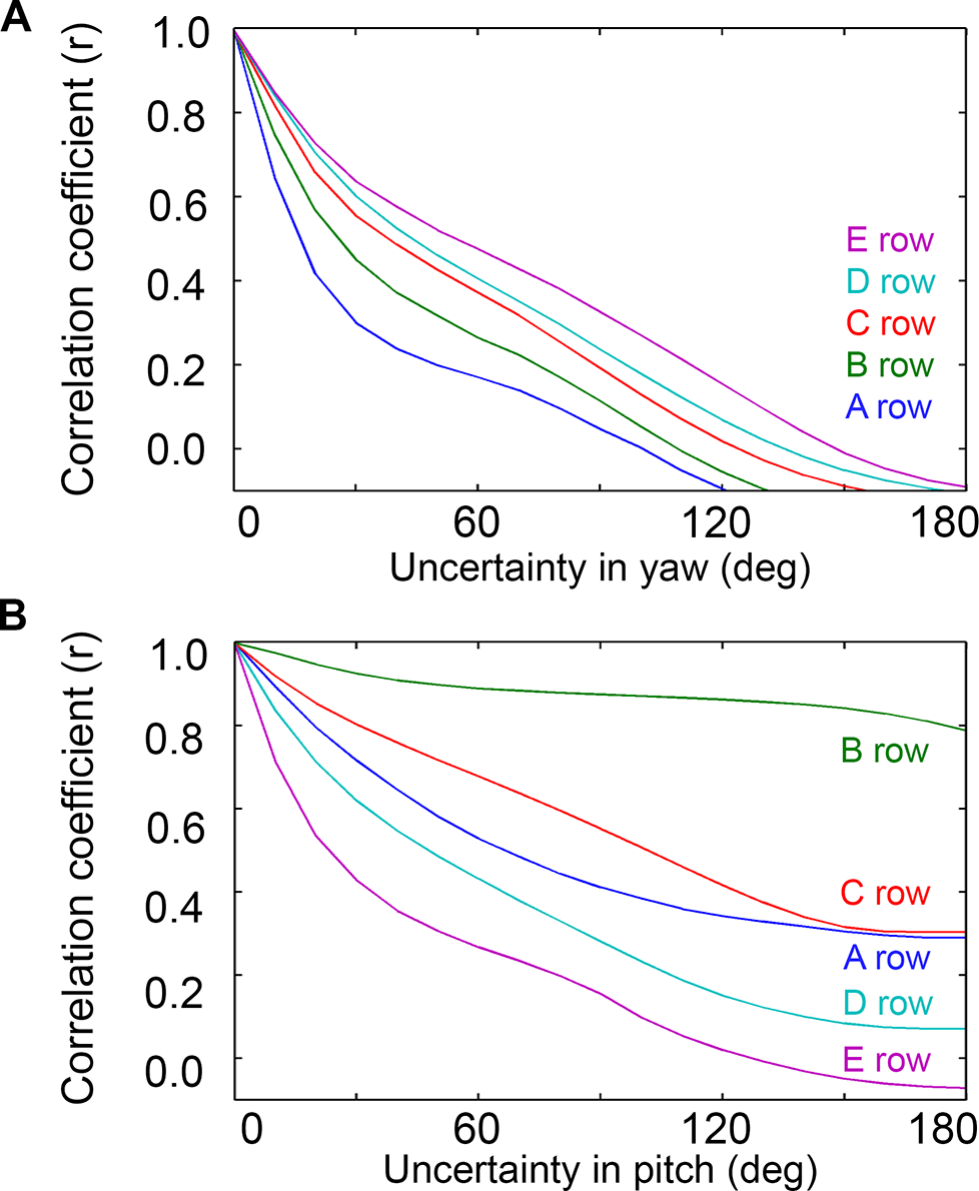
Correlation between θ_impact_ and distance as a function of uncertainty in yaw and pitch. Both analyses include whisking contacts only. **A.** For all rows, the correlation between θ_impact_ and distance falls off rapidly as the uncertainty in yaw increases. The value of 0° on the x-axis corresponds to the yaw being known exactly, 180° corresponds to the yaw being allowed to take on any value. Pitch is held at a constant value of 0°. **B.** The correlation between θ_impact_ and distance falls off differently for each row as uncertainty in pitch increases. The B and C rows show a greater robustness to uncertainty in pitch than do the exterior (A, D, and E) rows. The value of 0° on the x-axis corresponds to the pitch being known exactly, 180° corresponds to the pitch being allowed to take on any value. Yaw is held at a constant value of 0°.

The analysis for yaw (Fig 10A) showed that the correlation between θ_impact_ and distance dropped off dramatically for all rows if yaw is not known to within 30° (r < 0.66). In fact, as the uncertainty in yaw increases even more, the correlation actually becomes negative. Behaviorally, however, this uncertainty may not pose a problem for the rat, as Fig 6 has shown that yaw could easily be determined based on the difference in the number of right and left whiskers touching the surface.

The analysis for pitch (Fig 10B) shows that the mappings for the interior B and C rows—but not the A, D or E rows—result in a correlation between distance and θ_impact_ that is robust to changes in head pitch. When the uncertainty is 0° (i.e., pitch is known exactly), all rows show a very strong correlation between θ_impact_ and distance, as expected. As the uncertainty is increased, however, the correlation decreases differently for each row. The correlation for the B-row, in particular, remains remarkably strong for all amounts of uncertainty, even out to 180°, when pitch can take on any value. The C-row shows the second strongest correlation in the presence of uncertainty, with correlation coefficients remaining considerably larger than the exterior rows until ~90° uncertainty in pitch.

In summary, if the rat knows its orientation relative to the surface with high precision, θ_impact_ from any whisker could be used to determine the distance to the surface. If the pitch is not well known, as would be expected when a rat approaches a novel object, then θ_impact_ from the B and C rows will provide the rat with a far more reliable estimate of distance than will the A, D, or E rows.

### Rostral vibrissae across all rows contact only at very short distances

**Fig 11:**
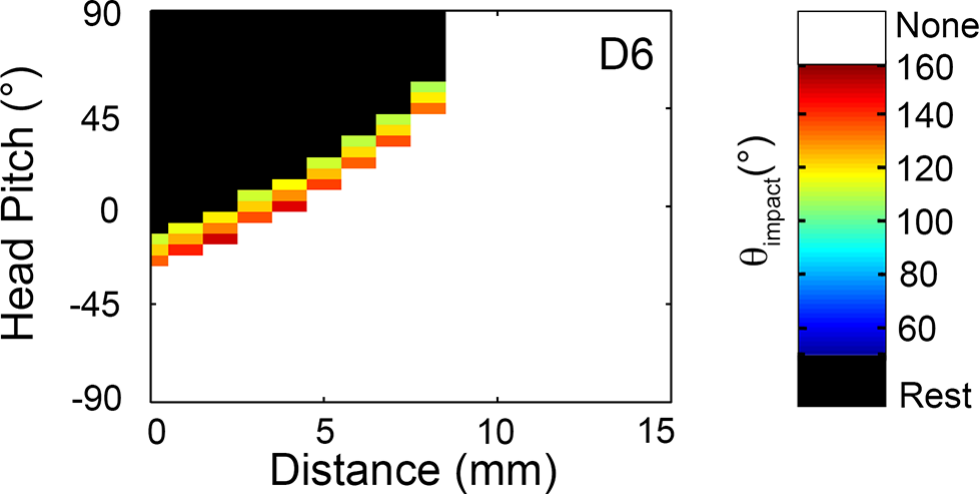
Typical contact pattern of a rostral vibrissa. The D6 vibrissa, like other small rostral vibrissae, tends to make resting-contacts with objects within 10mm of the rat’s snout. Whisking contacts are rare and tend to occur at only values of θ_impact_ above 100°.

The rostral-most vibrissae, such as D6, are the smallest vibrissae in the array. As a result, they can make contact only with the nearest of surfaces and exhibit a very limited pitch-distance mapping. For example, the B5, C5, C6, and D6 whiskers are limited to contacts within a distance range of 10mm when yaw = 0°. As shown in Fig 11, these vibrissae only rarely come into contact with an object during whisking; instead, if contact with an object occurs, it is almost always in the form of a “resting-contact.” Additionally, the values for *θ*_*impact*_ are extremely high, that is, these whiskers are able to make whisking-contact only when the array is protracted extremely far forward.

Fig 11 is likely to represent an underestimate of whisking contacts, because during natural behavior head movements will have a strong effect on vibrissal contact with a surface. Although head movement has an effect on the number of whisking contacts for all vibrissae, the effect is likely to be particularly pronounced for the rostral whiskers because the range over which whisking contacts occurs is so small.

### The vibrissae of the Greek column have particularly long distance reaches to the side

Visual inspection of Figs 6 and 7 indicates that the θ_impact_ mappings for the vibrissae of the Greek column smoothly fit the trend of the vibrissae of their respective rows. Because these whiskers represent the extreme limits of the row-wise trends, however, they also exhibit some unique mapping characteristics.

**Fig 12:**
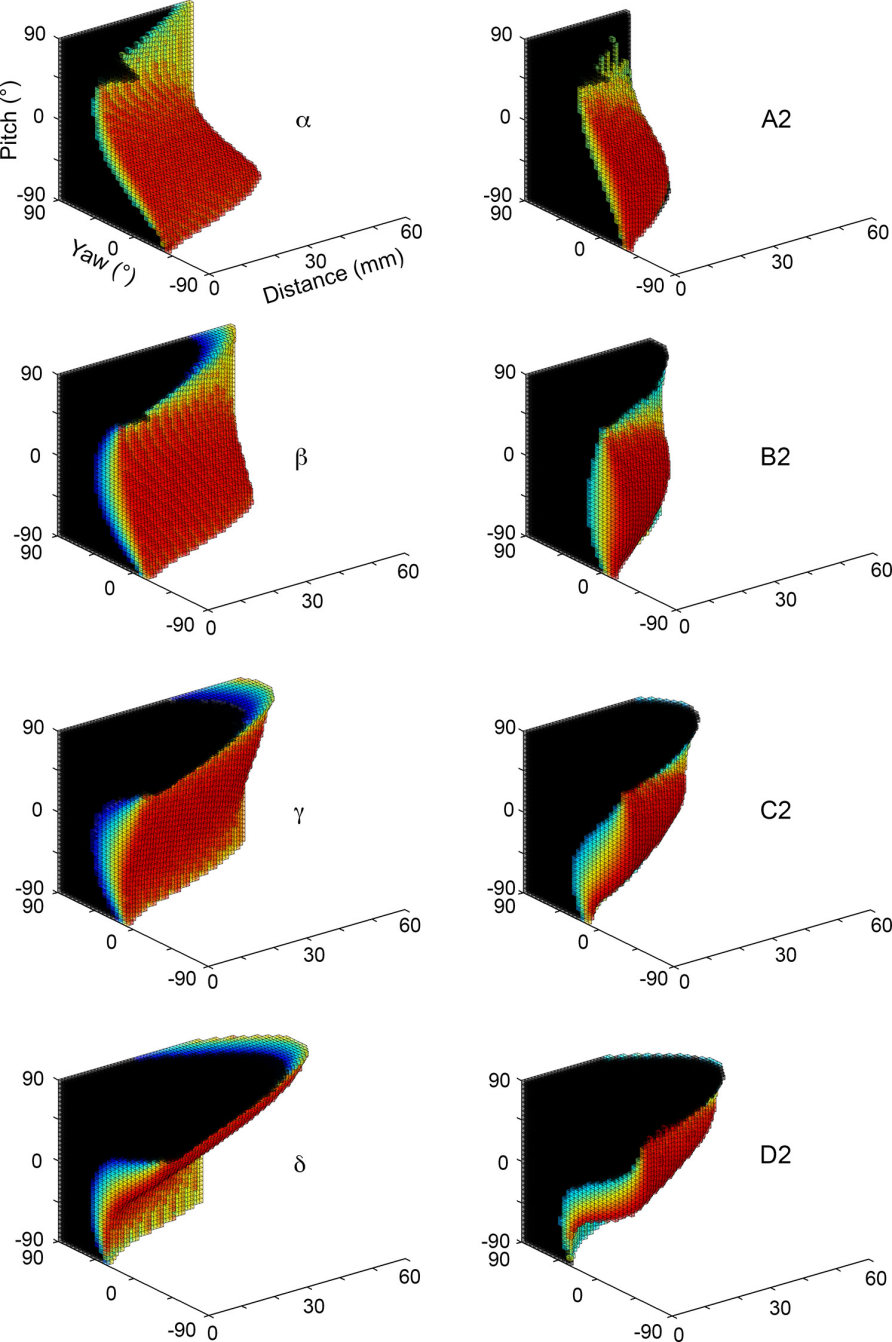
Vibrissae of the Greek column exhibit long reaches to the side. The vibrissae of column 2 (right) show convex mappings when yaw and pitch are near zero (θ = 0°, ϕ = 0°), while the vibrissae of the Greek column (left) are concave near this region. This means that the whiskers of the Greek column cannot touch the surface if the rat faces it symmetrically with a level head. The rat must either pitch its head up or down, or turn its head to the side. In all subplots black indicates a resting-contact, white indicates no contact, and the color scale indicates the value of θ_impact_. To permit visual comparison across subplots, the values of θ_impact_ have been normalized between 0 (dark blue) and 1 (dark red).

Fig 12 compares the distance-pitch-yaw mappings for the four vibrissae of the Greek column to the mappings for the whiskers of column 2. Because they are some of the longest whiskers in the array, the vibrissae of the Greek column can obviously reach to larger distances than can the column 2 whiskers. The figure also shows that, as for all the A and D row whiskers (Fig 7), the alpha and delta whiskers are close to mirror images of each other in pitch.

As previously demonstrated for the B3 whisker (Fig 9), Fig 12 shows that the column 2 whiskers all exhibit convex mappings when pitch and yaw are near zero. In contrast, the Greek whiskers tend to exhibit mappings that are concave near values of pitch = 0, yaw = 0. The functional consequence of this curvature difference is that—except for distances 1 mm or less from the surface—the alpha, beta, and gamma whiskers are never in contact at (θ = 0°, ϕ = 0°), while the all of the column 2 whiskers are. Delta is only in contact at (θ = 0°, ϕ = 0° for distances less than 15 mm.

Additionally, the mappings suggest that when the rat directly faces the surface with right-left symmetry (yaw = 0°), and a distance greater than 1mm, the alpha and beta vibrissae cannot whisk to touch the surface unless the head is pitched below -25°, the gamma whisker cannot touch the surface unless the head is pitched above 5°, and the delta whisker cannot contact the surface unless the head is pitched above -20°. Furthermore, when the rat’s head is level with the ground (pitch ≈ -20°), and the surface is greater than 1 mm away, the alpha, beta, and gamma whiskers are unable to touch the surface unless the rat’s side is turned towards the wall by 5° or more, or the angle of protraction is beyond the simulated range of 60°. These large protraction angles are described further in the last section on “extreme whisking.”

### Average θ_impact_ across rows and columns could be used to determine pitch and distance

The figures of the previous sections have indicated that the value of θ_impact_ from a single vibrissa alone does not permit unique identification of the distance-pitch configuration. The results have also shown, however, that the mappings between θ_impact_ and the pitch-distance configuration vary considerably across vibrissae. This variability could allow the rat to uniquely determine both pitch and distance based on values of θ_impact_ across the array.

We searched through all 83,509 distance-pitch-yaw configurations to determine which ones were associated with unique contact patterns of θ_impact_ across the two sides of the array. For every configuration, each whisker is labeled by its value of θ_impact_. Thus each configuration is represented by an array of 62 values corresponding to each whisker’s value of θ_impact_. A pattern is unique if that array of 62 values is different from all other 83,508 arrays.

Throughout this analysis, it is important to keep in mind that that the θ_impact_ contact patterns are directly generated by the kinematic simulation run at different (distance-pitch-yaw) configurations. Contact patterns that are “unnatural” will not be generated by the simulation. For example, a pattern with C2, C4, and C6 in contact with C1, C3, and C5 out of contact would never occur.

The search through all distance-pitch-yaw configurations showed that nearly a quarter (22.7%) of the configurations could be uniquely determined if θ_impact_ were known to within 10°. This percentage increases to 36.9% if θ_impact_ is known to within 5°. Close to half (49.7%) the configurations are unique if θ_impact_ is known to within 1°.

**Fig 13:**
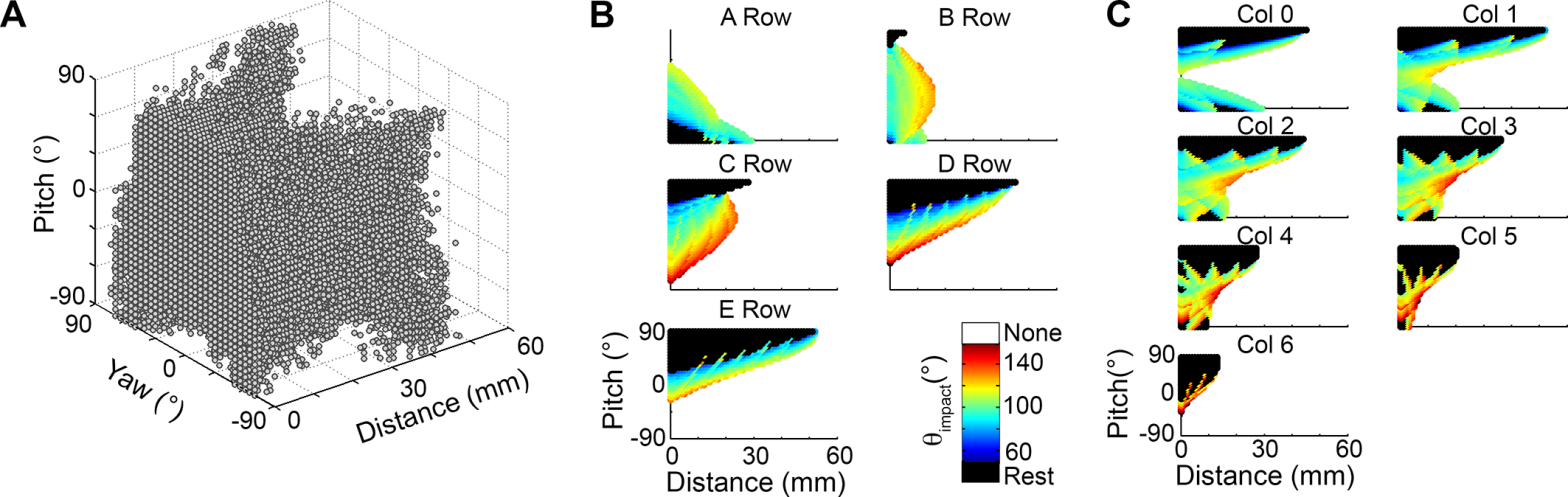
Values of θ_impact_ across the array constrain possible head configurations relative to the surface. **A.** A grey dot is placed at each distance-pitch-yaw configuration associated with a unique pattern of θ_impact_ across the array. Approximately 37% of the configurations can be uniquely determined if the values of θ_impact_ are known to within 5°. Note the non-unique region at distances less than 5mm. **B.** The value of θ_impact_ averaged within each row for different distance and pitch configurations (yaw = 0°). **C.** The value of θ_impact_ averaged within each column for the same configurations as in **B** (yaw = 0°).

Fig 13A shows the unique configurations when θ_impact_ is known to within 5°. An intriguing feature of Fig 13A is that many of the distance-pitch-yaw configurations cannot be uniquely determined for distances less than 5mm. At these short distances many of the vibrissae are in resting-contact with the surface, and the value of θ_impact_ therefore does not vary with configuration within that distance range. This result suggests that once the rat is very close to the surface, it must rely on information other than θ_impact_, such as mechanical variables, to extract information about distance to the surface and the pitch of its head relative to the surface.

The percentages listed above, and the data shown in Fig 13A, are based on an analysis that included both resting and whisking contacts. The percentage of unique configurations is much larger when the analysis is limited to include only those configurations that contain a minimum number of whisking contacts. If at least three vibrissae make whisking contact and θ_impact_ is known to within 5°, 24,954 out of 35,404 configurations (70.5%) are unique, and if θ_impact_ is known to within 1°, 33,636 out of 35,404 (95.0%) are unique. If θ_impact_ is known to 5°, all configurations can be uniquely determined if at least 9 vibrissae make whisking contact. If θ_impact_ is known to 1°, all configurations can be uniquely determined if at least 6 vibrissae make whisking contact.

The analysis of Fig 13A implicitly assumes that the rat has stored an extensive lookup table that matches the values of θ_impact_ across the array to the distance-pitch-yaw configuration. This is a memory-expensive solution. Alternatively, there are a myriad of heuristic methods that the rat could use to determine the angle of the surface relative to its head.

One possible heuristic follows from the values of θ_impact_ generated by averaging over rows and columns, shown in Fig 13B and 13C. Averaging across rows or columns provides tremendous robustness: even if a whisker is lost, damaged, or in the process of re-growing, the rat can still determine pitch and distance by using these trends instead of relying on precise values from each individual vibrissa. Fig 13B shows the average θ_impact_ across rows for all pitch-distance configurations, with yaw set to 0°. Notice how similar these are to the individual whisker mappings discussed earlier, suggesting that row-averaged values provide the rat with information about pitch and distance even if a specific whisker is missing.

When the same average is computed across columns, elements from every row contribute to form the “starburst” patterns shown in Fig 13C. For each column, there is a “hot” (large θ_impact_) region for pitch near 0°, demonstrating that large protraction angles are needed for the vibrissae to contact the surface. Additionally, the identity of the columns that exhibit contact provides important information about distance: for example, column 4 contact constrains the distance to within ~30mm; column 5 contact constrains the distance to within ~20mm; and column 6 contact constrains the distance to within ~10mm.

### Behavioral confirmation of predicted trends

**Fig 14:**
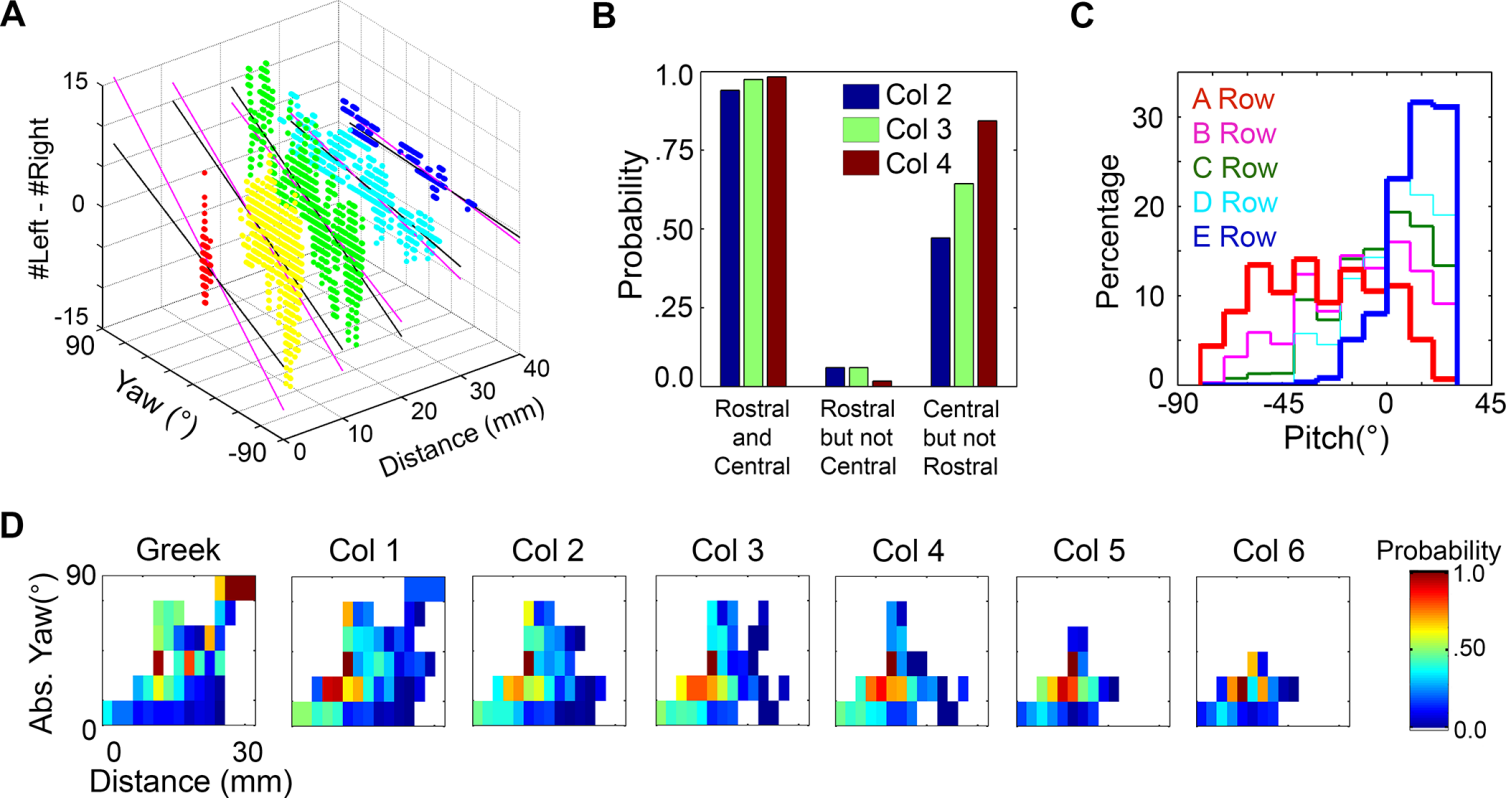
Behavioral data validate many predictions of the simulation. **A.** Behavioral data replicate the result of Fig 6B. The difference in the number of contacts between the left and right arrays provides information about yaw. Each data point corresponds to one msec in the behavioral data, and the data points have been color-coded by distance for visual clarity. The black lines are fit to the behavioral data and the magenta lines correspond to the predicted slope from Fig 6B (averaged across pitches). **B.** If a rostral vibrissa (Col 5, 6) is in contact with the surface, vibrissae of the central columns (Col 2, 3, 4) are almost guaranteed to be in contact. Similarly, if a rostral vibrissa in contact, it is rare that there is no contact by a central column vibrissa. Conversely, if a central vibrissa is in contact, the probability a rostral vibrissa being in contact is more moderate. **C.** Contact by vibrissae of the exterior rows (row A, D, E) provides information about pitch. The A row is more likely to be in contact for negative pitch values while the D and E row are more likely to be in contact for positive pitch values. The interior B and C rows show distributions intermediate between those of the A and E rows. **D.** The Greek vibrissae are able to contact the wall for large yaws and at large distances. The color of each box corresponds to the number of msec in which contact was observed for a given configuration divided by the total number of msec the rat spent in that configuration.

Data from a behavioral experiment in which naïve rats explored a flat glass wall confirmed four key predictions of the simulation results:

**The number of right-left vibrissae in contact correlates with yaw:** Fig 14A can be directly compared with Fig 6B. The figure illustrates the difference in the number of left vibrissae and right vibrissae in contact with the wall as a function of distance and yaw. The data points are from the behavioral experiment, not simulation. The black line corresponds to the best fit to the behavioral data, and the magenta line corresponds to the slope predicted from Fig 6B (averaged over pitches). As predicted by the simulations, the difference between right and left contacts correlates well with yaw, and the trend becomes flatter as distance is increased.

**More central vibrissae can reach surfaces at larger distances than even the most rostral vibrissae:** The simulation results of Fig 11 show that the rostral-most (columns 5 and 6) vibrissae will contact the wall only at very small distances. Comparing Fig 11 with Figs 8 and 9 suggests that it would be unlikely for only the rostral vibrissae to be in contact without the more central vibrissae also making contact. Fig 14B validates this prediction during exploratory behavior. The figure shows that if a rostral vibrissa is in contact, a vibrissa from a central column (columns 2, 3, or 4) is almost guaranteed to be in contact. It is extremely rare that a rostral vibrissa is in contact without a central column whisker also making contact. In contrast, contact by a column 2, 3 or 4 vibrissa does not mean a rostral vibrissa is necessarily in in contact.

**The exterior rows (rows A, D, and E) provide key information about pitch:** Fig 14C shows that during exploratory behavior the A row (red) is more likely to be in contact for low pitch values, while the D (cyan) and E (blue) rows are more likely to be in contact for high pitch values. The distributions for the B and C rows are more skewed to the high-pitch values than predicted by simulation; this is a subject for future study.

**Vibrissae of the Greek column have particularly long reaches to the side:** Fig 14D shows a heat map in which the color corresponds to the behaviorally-measured probability of contact for each (distance, yaw) configuration. The probability of contact was calculated as the number of msec in which a vibrissa of that column was in contact, divided by the total number of msec that the rat spent in that configuration. The figure shows that the Greek vibrissae are able to reach large distances at large values of yaw. In fact, when the rat is in these orientations, there is almost always contact by one of the Greek vibrissae (probability > 0.8). Column 1 vibrissae are also able to make contact at some of these extreme configurations, but do so much less frequently. Central and rostral vibrissae have a more limited reach to the side.

### Extreme whisking: Functional groupings are robust to large changes in whisking kinematics

It is well known that the rat has many degrees of freedom to adjust whisking kinematics as it explores [6,14,15,25,30,31], but this kinematic variability is not yet well characterized. For example, extrinsic facial muscles move the mystacial pad during whisking, and these motions translate the whisker basepoints in ways that have yet to be quantified. In the present section we alter whisking kinematics in several extreme ways in order to develop an intuition for the extent to which the mappings and functional groupings are likely to be robust to these as-yet unmeasured kinematic variations.

#### Variation 1: Retraction from rest and very large protractions

In all results presented so far, protractions have always started from biomechanical rest, the angle at which none of the rat’s facial muscles are contracted. It is well-documented, however, that the rat can retract its whiskers further caudal than mechanical rest. In addition, the simulations presented above have generally assumed that all whiskers protract through the same amplitude. But it is also well known that during some of the rat’s exploratory behaviors the central and caudal vibrissae can protract through larger angles than the rostral vibrissae [10,14,25].

**Fig 15:**
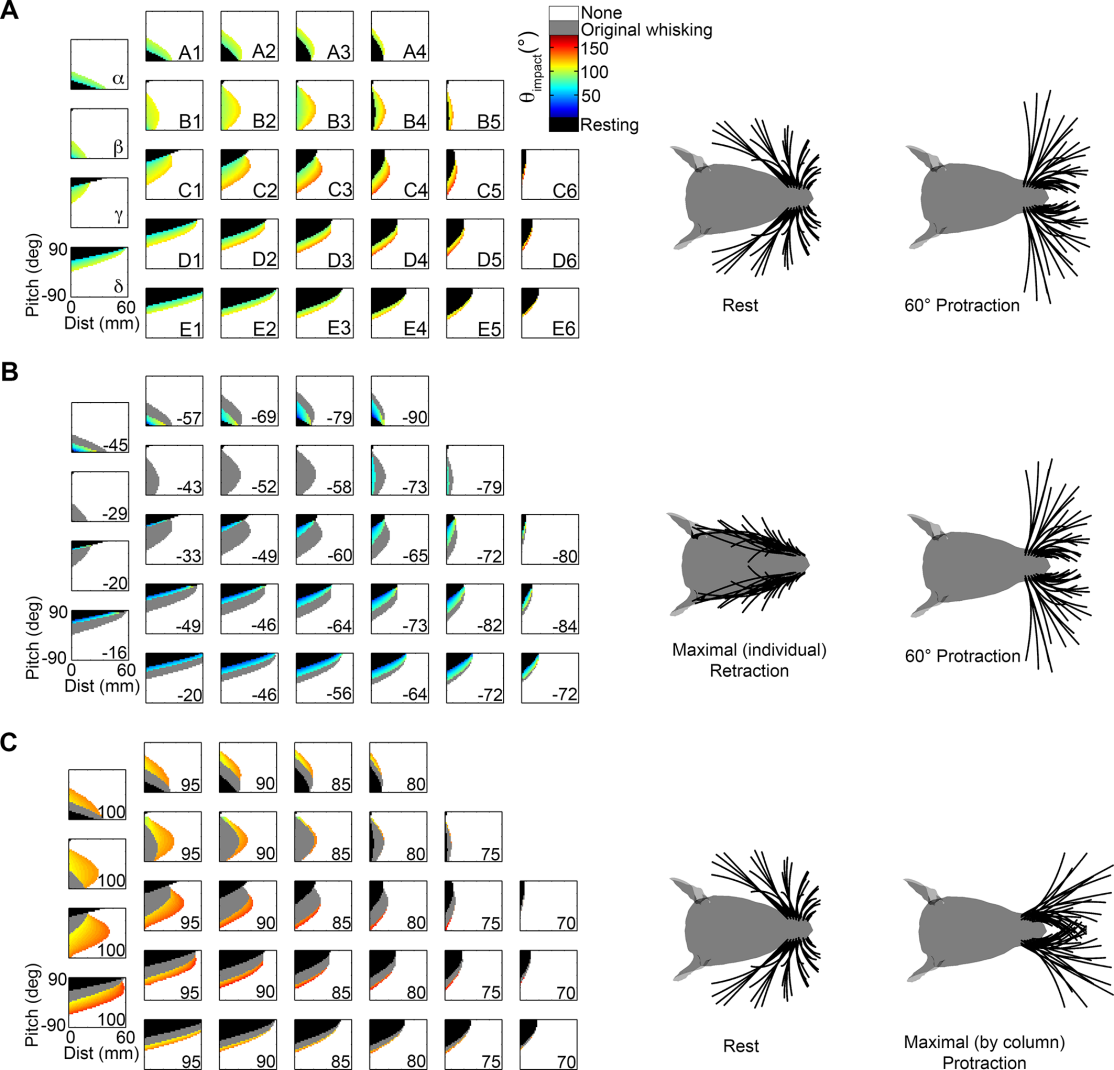
Mappings that include extreme retraction and protraction angles. Each mapping shows the distance of the head to the wall on the x-axis and the head pitch on the y-axis, with color indicating the value of θ_impact_. The yaw of the head is always held fixed at 0°. The two figurines to the right of the mappings illustrate the angular ranges of the whiskers spanned in the mappings. **A.** The original distance-pitch mappings for each of the 31 vibrissae. Each whisker starts at its biomechanical rest and is simulated to protract 60°. This figure is identical to Fig 7, except that the color scale has been extended to match the range of θ_impact_ shown in parts (B) and (C). **B.** Each vibrissa starts from its fully retracted position and is protracted 60° past its biomechanical rest position. Configurations that produce resting contacts in these mappings as well as the original mappings (from rest to 60°, Fig 15A) are shown in black. Configurations that produce whisking contacts in both mappings are shown in gray. Configurations that were resting contacts in the original mappings but have now become whisking contacts are colored according to θ_impact_. **C.** Each whisker starts from its biomechanical rest angle and protracts by the angle indicated in column 6 of Table 4, to its full protraction. As in subplot (B), resting contacts maintained from the original mappings are shown in black. Whisking contacts maintained from the original mappings are in gray. New whisking contacts are colored according to θ_impact_.

Simulations of these two conditions—full retraction and large protractions—revealed that the mappings and functional groupings are quite robust to these changes. Results are shown in Fig 15B and 15C with the original mappings displayed in Fig 15A for reference. The color-scale is the same in all three subplots of Fig 15, but has been altered slightly from Fig 7 to accommodate the larger range of θ_impact_ values observed in this analysis.

The case of full retraction is illustrated in Fig 15B. In this figure, each of the 31 vibrissae is retracted to lie flat against the face to its fully retracted angle θ_FR_, and then protracted to 60° past its resting angle. Each whisker has a unique θ_FR_, determined by the angle at which the whisker would begin to penetrate the head, and (as in all previous simulations) each whisker has a unique resting angle, θ_rest_. These angles are shown in the columns two and three of Table 4, respectively. Unsurprisingly, the full-retraction mappings shown in Fig 15B are nearly identical to the original mappings of Fig 15A. The additional retraction encompassed in Fig 15B merely changes some resting contacts to whisking contacts. These small changes are the only non-grayscale regions of Fig 15B.

**Table 4:** Simulated angular positions and amplitudes for full retraction and large protractions.

Whisker identity	(Position) θ_FR_	(Position) θ_rest_	(Position) θ_FP_	(Amplitude) θ_rest_ - θ_FR_	(Amplitude) θ_protraction_ ≡ θ_FP_ - θ_rest_	(Amplitude) θ_FP_ - θ_FR_
**α**	22.9	67.9	167.9	45	100	145
**β**	23.5	52.5	152.5	29	100	129
**γ**	18	48	148	30	100	130
**δ**	37.4	53.4	153.4	16	100	116
**A1**	16	73	168	57	95	152
**B1**	18.8	61.8	156.8	43	95	138
**C1**	24.8	57.8	152.8	33	95	128
**D1**	39.4	62.4	157.4	23	95	118
**E1**	48	68	163	20	95	115
**A2**	9.7	78.7	168.7	69	90	159
**B2**	19.4	71.4	161.4	52	90	142
**C2**	20.4	69.4	159.4	49	90	139
**D2**	25.7	71.7	161.7	46	90	136
**E2**	29.3	75.3	165.3	46	90	136
**A3**	5.6	84.6	169.6	79	85	164
**B3**	23.1	81.1	166.1	58	85	143
**C3**	20.2	80.2	165.2	60	85	145
**D3**	17.2	81.2	166.2	64	85	149
**E3**	26.9	82.9	167.9	56	85	141
**A4**	0.6	90.6	170.6	90	80	170
**B4**	17.8	90.8	170.8	73	80	153
**C4**	25.8	90.8	170.8	65	80	145
**D4**	17.8	90.8	170.8	73	80	153
**E4**	26.6	90.6	170.6	64	80	144
**B5**	21.4	100.4	175.4	79	75	154
**C5**	29.4	101.4	176.4	72	75	147
**D5**	18.2	100.2	175.2	82	75	157
**E5**	26.3	98.3	173.3	72	75	147
**C6**	31.9	111.9	181.9	80	70	150
**D6**	25.5	109.5	179.5	84	70	154
**E6**	33.7	105.7	175.7	72	70	142

All angular positions are represented in degrees relative to the rostral-caudal axis, with θ = 0° pointing caudal and θ = 180° pointing rostral. **Column 1:** The identity of the whisker. **Column 2:** The angle θ_FR_ is the angular position at which the whisker is fully retracted against the head. Any further retraction would cause a portion of the whisker to penetrate the head. **Column 3:** The angle θ_rest_ is the angular position of biomechanical rest. This is the angular position of the whiskers when none of the facial muscles are contracted. **Column 4:** The angle θ_FP_ is the angular position at which the whisker is fully protracted in the large-amplitude simulations. **Column 5:** The magnitude of the angular difference between the maximum retracted position and the whisker’s resting position. The mappings corresponding to this range are shown in Fig 15B. **Column 6:** Protraction amplitudes (θ_protraction_) for simulations in which the central and caudal vibrissae were allowed to move through larger angles than the rostral-most vibrissae. The mappings corresponding to this range are shown in Fig 15C. **Column 7:** The full angular range covered, from θ_FR_ to θ_FP_.

The mappings that emerge when central and caudal vibrissae are allowed to protract through larger angles than the rostral vibrissae are illustrated in Fig 15C. In this simulation, the column 6 vibrissae were protracted through an angle of 70° and each sequentially more caudal column was protracted an additional 5°, so that the whiskers of the Greek column were protracted 100° from their resting angles. The protraction angle (θ_protraction_) for each whisker is listed in column 6 of Table 4. Note that θ_protraction_ is measured from θ_rest_. Again, the general shape of the mappings changes very little. The extra protraction simply results in additional “layers” of color beyond the original contacts (shown in gray). Obviously, the more caudal vibrissae show the greatest additions to their mappings, because they were protracted the furthest past the original mappings. Beta, gamma, C1, and B1 now have significantly more reach in front of the face, as seen in the new peak near small pitch values.

#### Variation 2: Mystacial pad movements that translate the basepoints in three dimensions

**Fig 16:**
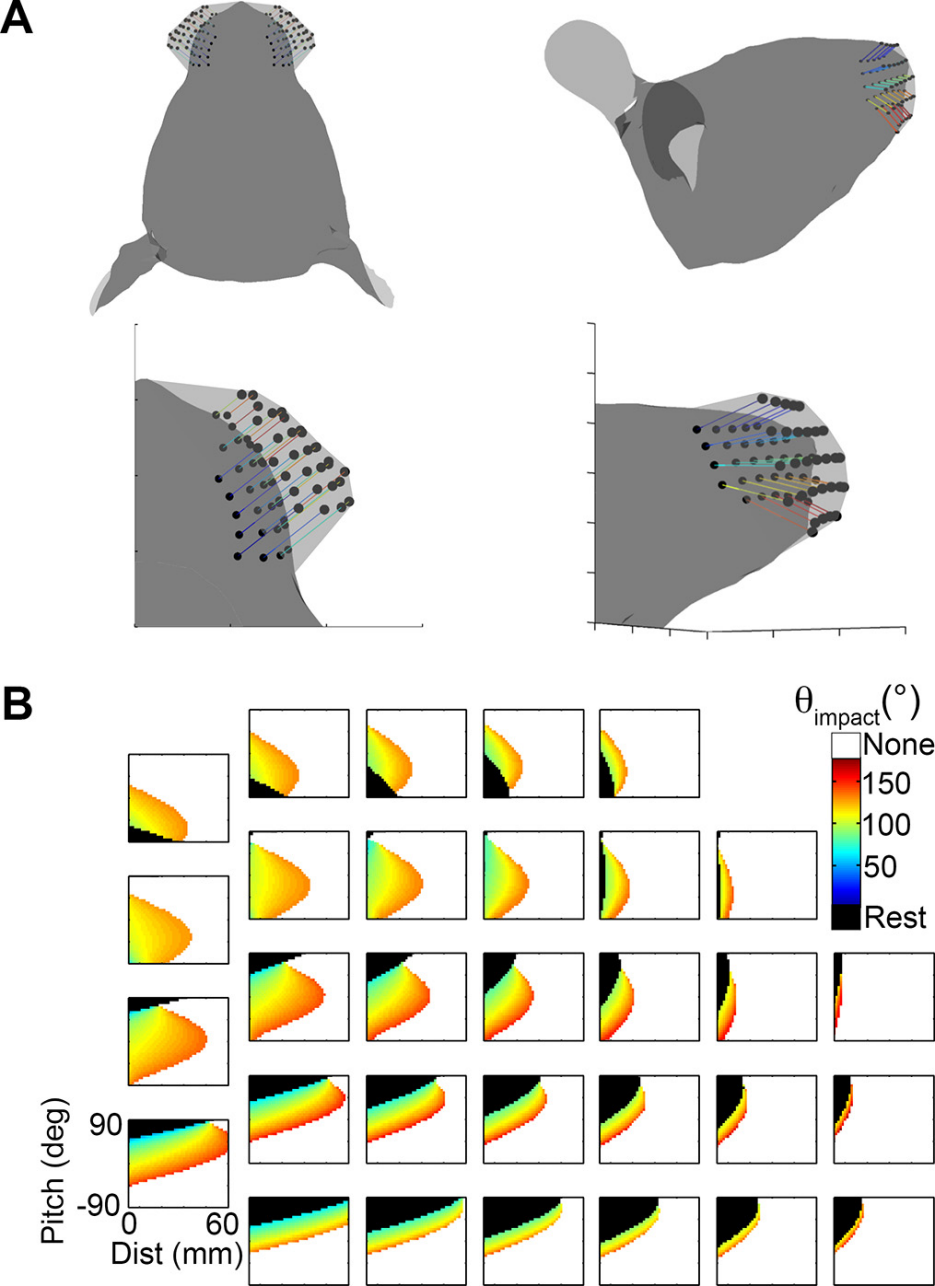
Effects of 3D basepoint translation. **A.** Vibrissal basepoints were translated by the amounts shown in Table 5 over the course of the protraction values shown in column 6 of Table 4. (Left) The translations of the basepoints are shown in a top-down view. (Right) The translations of the basepoints are shown in a side-on view. **B.** Basepoint translations have some small effects on the details of the mappings (compare with Fig 15C) but do not alter the functional groupings.

The rat can significantly alter the shape of its mystacial pad during whisking, which can generate large motions of the whisker basepoints. To examine the effect that this motion might have on the functional groupings, we ran simulations in which the vibrissal basepoints were allowed to shift over the course of a large protraction. The description of basepoint motion from Wineski (1983) [32] was used as a guideline; basepoints were translated in three dimensions an extreme amount (up to 5.17 mm) over the course of a simulated protraction. The basepoints moved forward and closer together in the anterior direction, while the mystacial pad simultaneously “puffed out,” like inflating a balloon. These motions are graphically depicted in Fig 16A, and the magnitudes of the basepoint displacements are provided in Table 5.

**Table 5:** Translations of the whisker basepoints during an extreme protraction.

	Greek	Col 1	Col 2	Col 3	Col 4	Col 5	Col 6
**A Row**							
Rostral-Displacement	3.17	2.87	2.57	2.29	2.03		
Lateral-Displacement	3.17	2.87	2.57	2.29	2.03		
Dorsal-Displacement	2.57	2.57	2.57	2.57	2.57		
***Total Distance***	***5*.*17***	***4*.*80***	***4*.*45***	***4*.*14***	***3*.*86***		
**B Row**							
Rostral-Displacement	3.17	2.87	2.57	2.29	2.03	1.79	
Lateral-Displacement	3.17	2.87	2.57	2.29	2.03	1.79	
Dorsal-Displacement	1.29	1.29	1.29	1.29	1.29	1.29	
***Total Distance***	***4*.*67***	***4*.*25***	***3*.*86***	***3*.*49***	***3*.*15***	***2*.*83***	
**C Row**							
Rostral-Displacement	3.17	2.87	2.57	2.29	2.03	1.79	1.56
Lateral-Displacement	3.17	2.87	2.57	2.29	2.03	1.79	1.56
No DV Displacement	0.00	0.00	0.00	0.00	0.00	0.00	0.00
***Total Distance***	***4*.*49***	***4*.*05***	***3*.*64***	***3*.*24***	***2*.*87***	***2*.*53***	***2*.*20***
**D Row**							
Rostral-Displacement	3.17	2.87	2.57	2.29	2.03	1.79	1.56
Lateral-Displacement	3.17	2.87	2.57	2.29	2.03	1.79	1.56
Ventral-Displacement	-1.29	-1.29	-1.29	-1.29	-1.29	-1.29	-1.29
***Total Distance***	***4*.*67***	***4*.*25***	***3*.*86***	***3*.*49***	***3*.*15***	***2*.*83***	***2*.*56***
**E Row**							
Rostral-Displacement		2.87	2.57	2.29	2.03	1.79	1.56
Lateral-Displacement		2.87	2.57	2.29	2.03	1.79	1.56
Ventral-Displacement		-2.57	-2.57	-2.57	-2.57	-2.57	-2.57
***Total Distance***		***4*.*80***	***4*.*45***	***4*.*14***	***3*.*86***	***3*.*60***	***3*.*39***

All values are in mm. Protraction angles used correspond to those of column 6 of Table 4. Values for rostral and lateral displacements were chosen by ensuring that the most rostral column of whiskers (Col 6) moved a little over 1.5 mm rostrally over a 70° protraction. The basepoints of each other column of whiskers were then simulated to translate incrementally more than those in its rostral column-neighbor such that Greek vibrissae translated just over twice as far as the Col 6 vibrissae. The magnitude of the dorsal/ventral displacements was chosen to be maximal for the A and E rows but in opposite directions, imitating the “puffing-out” of the mystacial pad. The magnitude of the dorsal/ventral displacement dropped off linearly to zero at the C row.

Fig 16B illustrates the results of this large basepoint motion when combined with the large protraction angles of Fig 15C. The differences compared to no basepoint movement are so small that they are difficult to observe by eye. Quantitative analysis showed that, unsurprisingly, all whiskers had a slightly further reach (particularly evident for the caudal whiskers), and that the values of θ_impact_ were all correspondingly lower (less than 5° change). Most importantly, however, the functional groupings remain unchanged. Thus, although basepoint motions will have important effects on the timing of contacts as well as the spatial location of contact on an object, they are unlikely to significantly alter the functional groupings described in this paper.

#### Variation 3: Effects of whisker length, intrinsic curvature, roll, and elevation

All simulations so far have been based on the equations for whisking kinematics derived from Knutsen et al., 2008 [6] (Table 2). During different types of exploratory behavior, however, it is possible that these whisking kinematics may be considerably altered. For example, the relationship between θ_protraction_ and roll and elevation might change for extreme values of θ_protraction_. We can examine extreme versions of these alterations to obtain an intuition for the extent to which the functional groupings are likely to be maintained.

Full 3D mappings were computed for the C2 vibrissa under seven different parameter variations: whisker length was increased by 25% or decreased by 25%; the intrinsic curvature of the whisker was doubled or set to zero (straight whisker); roll was eliminated, elevation was eliminated, and both roll and elevation were eliminated. C2 was allowed to protract through 90° from rest (consistent with the value in Table 4, column 6).

**Fig 17:**
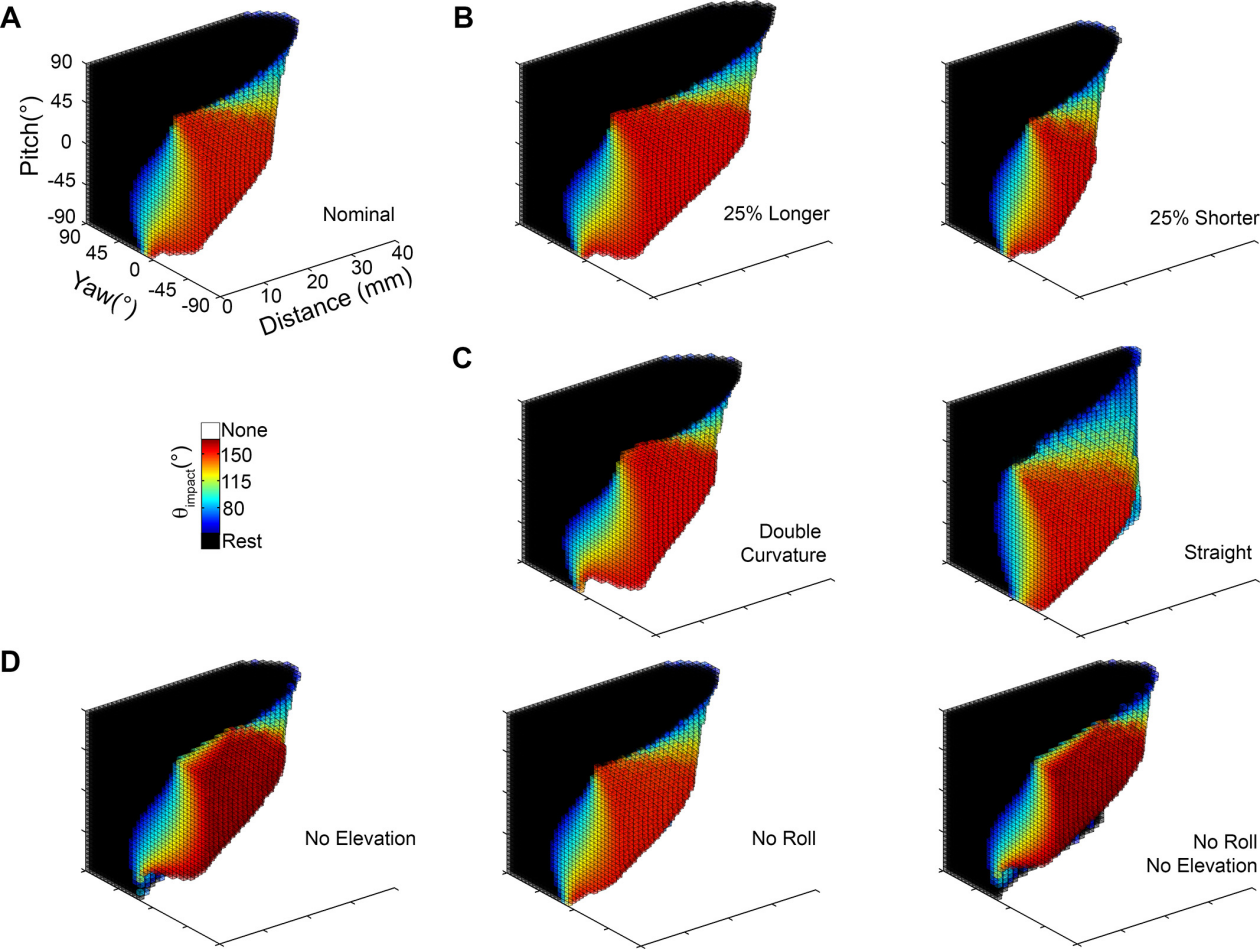
Effects of whisker length, intrinsic curvature, roll, and elevation on mappings for the C2 whisker. **A.** Original mapping. Seven changes were made to the C2 vibrissa and its kinematic equations: **B.** length was increased and decreased by 25%, **C.** intrinsic curvature was doubled or the whisker was made completely straight while retraining the same total length, and **D.** kinematic equations were modified to completely eliminate elevation, roll, and both roll and elevation. None of these seven alterations to the parameters alter the overall characteristics of the mappings or the functional groups.

Changing the length had the obvious effect of stretching and shrinking the mapping along the distance-axis compared to nominal (compare Fig 17A and 17B). When intrinsic curvature is doubled (Fig 17C) the mapping retains its characteristic shape with a slight increase in the steepness of the mapping for very low pitch values and extreme angles of protraction. This is seen in Fig 17C as an increase in the steepness of the red region near the bottom of the mapping. Conversely, making the whisker completely straight tends to flatten the mapping and to increase contacts for negative values of yaw. In all four of these cases, however, the basic features of the mapping remain intact.

When elevation is completely eliminated (Fig 17D) the rat has to pitch its head higher in order for contact to occur, thus the mappings shift towards higher values of head pitch. In turn, larger values of θ_impact_ are generally required for contact to occur. Elimination of roll has less of a pronounced effect, but in general results in contact at smaller values of θ_impact_. Eliminating both roll and elevation results in a mapping intermediate between removal of roll and elevation independently, quite similar to the original mapping of Fig 17A.

Collectively, the results of Figs 15–17 illustrate that although changes to whisker geometry and whisking kinematics do affect the mappings (as, indeed, must occur), even dramatic alteration to kinematics are unlikely to substantially shift the functional groupings.

## Discussion

### The statistics of vibrisso-tactile natural scenes

In the visual system, natural scene statistics are generally considered the prior distribution from which the eye samples to acquire information about the world [33]. These statistics generally include the spatial frequencies in the scene, often (though not always, see [34]) neglecting the effects of eye movements.

In the somatosensory system, it is more challenging to dissociate the sensory and motor components required to uniformly sample the statistics of the environment than it is for the visual system. Rats actively use their vibrissae to follow walls, monitor their relationship with the ground, and orient towards and explore novel objects [1,2,31,35,36]. Identifying the contact patterns for each whisker and the extent of each whisker’s reachable space would be nearly impossible with behavioral experiments, but simulations permit a comprehensive and systematic investigation of the rat’s search space. The results shown here begin to identify the vibrisso-tactile ranges available to the rat, and the relationships between array-wise contact patterns and surfaces in the environment.

These results thus provide some of the first quantitative insights into natural tactile scenes for the vibrisso-trigeminal system. A complete description of vibrisso-tactile natural scenes would include the forces and moments at the base of each whisker during exploration of environments containing a wide variety of curvatures and textures. The geometric (θ_impact_) quantifications shown in the mappings of this study are the first step on the way to this more complete description.

These mappings identify which whiskers can make contact with a flat surface, and the protraction angles at which those contacts will occur. Whiskers that show similar mappings can be thought of as sharing probability distributions of contact. Figs 7 and 13B reveal that the mappings are more similar by row than by column. Thus the row-wise structure that characterizes the receptive fields of many central neurons in the trigeminal system seems likely to reflect the row-wise structure of the contact patterns of the whiskers.

### Robustness of the functional groupings and the effect of head movements

The mappings revealed by the simulations of the present study (Figs 5–13) should be interpreted to represent broad principles that underlie rat exploratory behavior, not rigid constraints on vibrissal-object contact patterns. This study does not aim for precise values of θ_impact_, but rather to identify trends in θ_impact_ across surface positions and orientations, and across vibrissae in the array. The sensitivity analysis (see Methods) as well as the extreme variations added in the last section of results ensure that the functional groupings are generally robust to uncertainties in array morphology and whisking kinematics.

The demonstration that the functional groups are robust to high degrees of variation is particularly important given that the kinematics of the mystacial pad and its resulting effects on the location of the whisker basepoints during whisking have not yet been quantified. Mystacial pad motion may change substantially during different types of whisking behavior, and this could have large effects on the positions of whisker basepoints and the resulting whisking kinematics. Although Figs 15–17 demonstrate that geometric or kinematic variations are unlikely to change the functional groupings substantially, these variations do change the exact shape of the mappings. These changes will clearly be important when modeling contact timing in the future.

In addition to mystacial pad movements, head movements also play a key role in whisking behavior [2,14,31,32,35]. Although head movements will have a large effect on vibrissal-object contact patterns, they will not directly affect the mappings shown in the present study. The mappings are invariant to head movement because they are constructed in head-centered coordinates. As the head moves, however, its position and orientation relative to the surface will change, and so thereby the values of θ_impact_. Head movements are thus represented as transitions within a mapping (i.e., from one distance-pitch-yaw configuration to another). As an obvious example, if the head translates closer to a wall during a protraction, the value of θ_impact_ will be reduced by an amount related to the distance the head has traveled.

### Functional groupings of vibrissae and implications for behavior

Previous studies have already provided evidence for functional distinctions between rostral and caudal vibrissae [10,32,37]. For example, animals often use their rostral-most whiskers to maintain object contact while actively touching the object with the larger, more caudal whiskers [10,32]. The present study supports and extends these earlier results, strongly suggesting that vibrissae are functionally grouped across the array to differentially support contact with surfaces at different orientations relative to the rat, as follows:

*When the rat directly faces a surface with right-left symmetry and its head approximately level*, the central vibrissae will be able to contact a more distant surface than will the rostral or caudal vibrissae. Additionally, the B and C row vibrissae will contact the surface in a manner such that θ_impact_ provides information about distance to the surface. Fig 5 illustrates the somewhat surprising result that the smallest reachable distance is centered around pitches and yaws close to 0°, right in front of the rat’s nose; a wall directly in front of the rat must be closer than a wall to the side or below the nose in order to be detected. Thus to explore a wall directly in front of the nose the rat would either have to protract its whiskers through a larger angle than is typically observed during locomotion [38,39], move its head closer to the wall, or both.

*When the rat locomotes forward*, most of its ventral vibrissae will be in contact with the ground [38,39]. However, multiple laboratories have documented that rat tactual exploratory behavior, including during locomotion, is characterized by periodic “head-dabs,” approximately synchronized to the whisking cycle [2,30,38,40]. These head dabs press the short micro-vibrissae against surfaces and may allow the olfactory system to acquire chemosensory information. Fig 5C shows that in order to maximize the reachable distance in the direction of forward locomotion, the rat would need to turn its head to the side. We therefore suggest that the magnitude of the medio-lateral component of head dabbing behavior during locomotion reflects a compromise between sensory coverage and locomotor ease.

*When the rat faces an object and tips its head up or down*, the exterior vibrissal rows will provide information about the relative pitch of the surface. The vibrissae of the A row will make contact when the head is pitched strongly downward. The vibrissae of the D and E rows will make contact when the head is pitched upward, corresponding to orientations observed during rearing and typical locomotor behaviors, and consistent with a role for these whiskers in ground-following [1]. Rearing behavior (corresponding to very large values of pitch) allows the rat to explore the wall as it does the ground, bringing numerous ventral vibrissae into contact. Rearing thus does more than simply increase the rat’s vertical exploration space: it enables contacts with the surface to be made at much larger distances than for a head-on pose.

*When the rat turns its right or left side towards a surface*, the difference between the total number of right and left whiskers in contact correlates with the yaw. Increasing numbers of caudal whiskers will be able to come into contact during a whisk. The vibrissal array’s maximum reach occurs for the Greek vibrissae, for large values of yaw. It has been suggested that the Greek vibrissae may have more evolutionary commonality with other facial hairs such as guard hairs [41]. Their limited range of whisking contacts (Fig 9) and larger reach oriented towards the side (instead of the front) of the face suggest they are likely to be particularly important in wall-following.

*When the rat makes contact with a surface at very small distances*, the angles of contact (θ_impact_) of the rostral vibrissae will provide very little information about the orientation of the surface (Fig 13). In fact, it is common to see more central vibrissae in contact with the wall while it remains out of reach for the column 5 or 6 vibrissae (Fig 14). The extremely limited reach of the rostral-most macrovibrissae would enable them to function quite similarly to the microvibrissae, helping to guide the mouth toward food or other objects of interest. Given that the value of θ_impact_ provides almost no information about object distance for the rostral whiskers, additional information such as forces and moments are likely to be used in object feature extraction [40]. This result complements an earlier study which found that the microvibrissae are pressed against small objects to aid in the determination of object shape [37].

### Correspondence between tactile functional groups and muscles of the mystacial pad

Notably, the functional groupings identified here appear to correspond closely to the muscle groups that activate different regions of the array [13,42–46]. Specifically, *M*. *nasolabialis superficialis* is tightly anatomically coupled to the A-row and its expected effect is to elevate these vibrissae during protraction [44]. *Pars orbicularis oris* of the *M*. *Buccinatorius* is expected to mediate ventrocaudal deflection of vibrissal rows C-E, and its geometry suggests a stronger effect on rows D and E [44]. Together, *pars media inferior* and *pars media superior* are likely responsible for the protraction and convergence of all rows, but their effects appear to influence only whiskers more rostral than column 1 [44]. This would differentiate the caudal-most vibrissae as a functional group. Finally, a very large number of muscles are tightly coupled just to the rostral-most vibrissae [42,43].

In some respects, the correspondence between previously-identified muscle groups and the tactile mappings found in this work is not surprising, because the simulated kinematic trajectories (obtained from behaving animals) clearly affect the mappings. When we simulated the contact patterns without elevation and roll, the mappings were altered (c.f., Fig 17) although the functional groupings remained unchanged. Additionally, roll and elevation affect the sensory volume of the array during a protraction [20]. Together, these results highlight the key importance of incorporating all three dimensions of whisking kinematics when quantifying the acquired tactile data.

### Simultaneous extraction of pitch, yaw, and distance

The present work clearly demonstrates that there are multiple, redundant methods by which the rat could uniquely determine the distance, pitch, and yaw to a surface. One theoretical possibility is that the rat could use a “look-up” table that relates 62 precise values of θ_impact_ to a particular configuration (Fig 13A). This solution is computationally costly, relies on the precision to which θ_impact_ is known, and is susceptible to error if whiskers are damaged or missing. It seems more likely that the rat exploits the functional groups identified here. The rat could obtain an estimate of yaw by monitoring the difference in contacts between the left and right arrays (Fig 6); comparing contacts in the A vs. D/E row provides an estimate of pitch (Fig 8); and the average θ_impact_ within B and C rows provides an estimate of distance (Figs 9 and 10). Subsequent whisks could refine the estimation, because as one parameter is constrained it improves estimates of the other two.

A challenge to this proposed approach will occur for surfaces located at very close distances, as would occur when a rat places its microvibrissae on an object [2,4,30,37,47]. At close distances, the mappings are dominated by resting contacts, which always (by definition) have the same value of θ_impact_. In the configurations dominated by resting contacts, θ_impact_ across the array does not provide enough information to distinguish between the configurations. Instead, the rat may use an alternate approach such as monitoring the magnitudes and directions of forces of contact [48–50].

### Implications for electrophysiology

Whiskers are unlikely to be interchangeable sensors: A key point of the present work is that morphological trends across the array produce groupings of vibrissae that exhibit distinct probability distributions for surface contact. If neurons are tuned to match the statistical distributions of peripheral input, as has been shown for many other sensory systems [51–53] then the receptive field structure of neurons associated with different vibrissae is likely to reflect these different functionalities.

**Fig 18:**
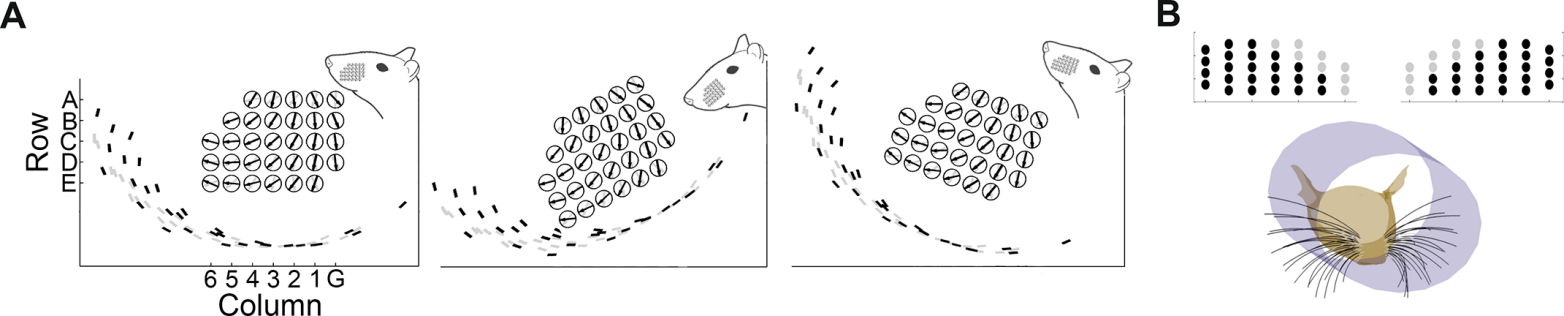
Head pitch, intrinsic curvature, and implications for simulation patterns. **A.** The intrinsic curvature for each vibrissa is shown relative to the head at a level pitch (left), the head pitched down (middle), and the head pitched up (right). Gray traces correspond to the direction of intrinsic curvature at rest and black corresponds to the direction of intrinsic curvature after a protraction of 60°. **B**. In a burrow most of the vibrissae are in resting contact with a surface, but the force exerted by the surface on the whiskers is in different directions for different rows.

These functional groupings are particularly informative when combined with earlier results showing that the intrinsic curvature of the whiskers varies relatively smoothly across the array, as illustrated in Fig 18A (left) [7,8,48]. Two key trends are apparent in the figure. First, neighboring whiskers tend to have similar orientations. Intrinsic curvature changes from concave-forward in the rostral region of the array to more concave-down in the caudal region. Second, the orientation at which the whiskers will make contact with a surface changes considerably more for the A, D, and E rows than it does for the B and C rows.

With these two trends in mind, the present work allows us to begin to make some predictions for receptive field structures and tentatively suggests some useful patterns of peripheral stimulation.

**Dorsal/ventral differences between the A/E rows:** The vibrissae of the A-row are most likely to make contact with a vertical surface in front of the nose when the head is pitched down; in contrast, the E-row vibrissae are more likely to make contact when the head is pitched up (Fig 8). In these orientations, A-row vibrissae will hit in a mostly concave-down orientation (Fig 18A, middle), while E-row whiskers will tend to contact largely concave-forward (Fig 18A, right). Experimenters may therefore wish to add a slight ventrally-directed component of motion to rostral-caudal stimulation of the A-row, and a slight dorsally-directed component to the E-row. Intriguingly, in a study of direction-tuning in rostral spinal trigeminal nucleus interpolaris, all neurons were found to be strongly tuned to stimulation in the dorsal direction, but a small group of neurons specifically associated with the A-row exhibited significant tuning to the ventral direction [54].

The mappings of Fig 8 also show that the rat will rarely experience simultaneous contacts on the A and E rows when exploring a flat surface. In contrast, when inside a burrow, numerous whiskers from all rows will be in contact, including both A and E (Fig 18B). However, the orientations of A and E vibrissae will be different. The A row will hit the “side” of the burrow, while the E row will hit the “floor.” To simulate the deflections observed in such a scenario, the A-row whiskers could be deflected back and forth in purely rostral-caudal pattern, while including a small steady-state dorsally-directed component to the E-row whiskers.

**Predictions for receptive fields of neurons associated with B and C row vibrissae:** The B and C rows exhibit the largest relative number of “whisking-contacts”, in which the vibrissae are not in contact with the surface at the start of protraction, but are brought into contact during the whisk. Furthermore, as shown in Fig 9, their value of θ_impact_ is strongly correlated with distance to the surface, independent of head pitch. Finally, as noted above, the roll of the whisker about its own axis is smallest for the B and C rows (the direction of the intrinsic curvature changes little from rest through a 40° protraction), so that the direction in which the whisker will be deflected by a surface is relatively independent of protraction angle. These vibrissae are thus most likely to have neurons with receptive fields that explicitly represent the value of θ_impact_, perhaps even performing a center-surround comparison of the value of θ_impact_ within a row.

**Sustained vs. transient stimulation:** As described above, all whiskers exhibit a significant number of “resting contacts.” The E and D rows make extensive resting contact with the ground, whiskers of the Greek column are often in resting contact with a side wall, and the rostral whiskers of columns 5 and 6 are in resting contact for the majority of their configuration space. Standard “ramp and hold” stimuli given in electrophysiological experiments typically involve a rapid deflection from rest, with a sustained plateau and then a rapid return to the resting position. It is possible that a more naturalistic stimulus for these whiskers would involve a transient deflection riding on a small sustained (DC offset) deflection. We anticipate that a higher fraction of slowly adapting neurons may characterize the receptive field structure for these whiskers, compared to the central whiskers of the A, B, and C rows.

### Conclusion

Some vibrissae on the rat’s face are already thought to serve highly specialized functions. For example, the supraorbital vibrissae are clearly important in protecting the eyes [32,55], and the vibrissal trident is important for determining running speed [39]. In contrast to these “special” vibrissae, vibrissae of the mystacial pad are often thought of as nearly-interchangeable sensors which, though varying in length and appearance, are essentially identical in function. Instead, the present work suggests that vibrissae in different regions of the array show specific strengths and means by which information about the environment can be obtained. In future work we aim to identify the behavioral strategies that the rat employs to exploit these functional groups, which in turn may shed light on how the neural circuitry has evolved around these natural tactile-scene statistics.
